# Maximal mid-expiratory flow is a surrogate marker of lung clearance index for assessment of adults with bronchiectasis

**DOI:** 10.1038/srep28467

**Published:** 2016-06-24

**Authors:** Wei-jie Guan, Jing-jing Yuan, Yong-hua Gao, Hui-min Li, Jin-ping Zheng, Rong-chang Chen, Nan-shan Zhong

**Affiliations:** 1 State Key Laboratory of Respiratory Disease, National Clinical Research Center for Respiratory Disease, Guangzhou Institute of Respiratory Disease, First Affiliated Hospital of Guangzhou Medical University, Guangzhou, Guangdong, China; 2 Department of Respiratory and Critical Care Medicine, First Affiliated Hospital of Zhengzhou University, Zhengzhou, Henan, China

## Abstract

Little is known about the comparative diagnostic value of lung clearance index (LCI) and maximal mid-expiratory flow (MMEF) in bronchiectasis. We compared the diagnostic performance, correlation and concordance with clinical variables, and changes of LCI and MMEF% predicted during bronchiectasis exacerbations (BEs). Patients with stable bronchiectasis underwent history inquiry, chest high-resolution computed tomography (HRCT), multiple-breath nitrogen wash-out test, spirometry and sputum culture. Patients who experienced BEs underwent these measurements during onset of BEs and 1 week following antibiotics therapy. Sensitivity analyses were performed in mild, moderate and severe bronchiectasis. We recruited 110 bronchiectasis patients between March 2014 and September 2015. LCI demonstrated similar diagnostic value with MMEF% predicted in discriminating moderate-to-severe from mild bronchiectasis. LCI negatively correlated with MMEF% predicted. Both parameters had similar concordance in reflecting clinical characteristics of bronchiectasis and correlated significantly with forced expiratory flow in one second, age, HRCT score, *Pseudomonas aeruginosa* colonization, cystic bronchiectasis, ventilation heterogeneity and bilateral bronchiectasis. In exacerbation cohort (n = 22), changes in LCI and MMEF% predicted were equally minimal during BEs and following antibiotics therapy. In sensitivity analyses, both parameters had similar diagnostic value and correlation with clinical variables. MMEF% predicted is a surrogate of LCI for assessing bronchiectasis severity.

The lungs function as an organ for ventilation and gas-exchange. In physiologic conditions, ventilation heterogeneity^1^,^2^ exists because of differential distribution of blood and air as consequences of gravity gradients. In chronic respiratory diseases such as bronchiectasis^3^,^4^, these conditions may be aggravated by mucus hypersecretion and plugging^5^, pulmonary infections^6^,^7^ and airway remodeling^8^. Indeed, ventilation heterogeneity has been common in bronchiectasis and reportedly associated with poor lung function and abnormality of chest high-resolution computed tomography (HRCT)^3^,^4^. Lung clearance index (LCI)^3^,^4^,^8^, a parameter reflecting ventilation heterogeneity, may more sensitively discriminate bronchiectasis from health than forced expiratory volume in 1 second (FEV_1_)^4^, the ‘gold standard’ of spirometric assessment. Compared with chest HRCT, LCI offered complementary significance for diagnosing primary ciliary dyskinesia^9^ and cystic fibrosis^10^ and might sensitively detect early-stage cystic fibrosis^11^,^12^.

Early changes in lung architectures may be detected with maximal mid-expiratory flow (MMEF), a spirometric parameter reflecting airflow of large and small airways^13^,^14^. Compared with healthy subjects, MMEF was significantly lower in bronchiectasis^4^,^15^. Theoretically, MMEF might more sensitively reflect small airway disorders than FEV_1_ because of small airway airflow limitation. Because MMEF could be conveniently derived from spirometry, which requires less patient’s cooperation, shorter testing duration and less complex instruments, it is tempting to postulate that MMEF could substitute LCI for assessment of bronchiectasis or offer complementary information on lung function impairment. This is important since some patients who could not perform acceptable maneuvers, and certain medical institutions may lack testing instruments for inert gas wash-out tests that frequently preclude measurement of LCI.

We sought to investigate: 1) the diagnostic performance of LCI and MMEF; 2) association between LCI and MMEF; 3) concordance and correlation with clinical parameters; and 4) changes in LCI and MMEF during exacerbation^15^,^16^. We also performed subgroup analyses (in mild, moderate and severe bronchiectasis as categorized using an integrated disease severity metric and radiologic severity score) to further validate these findings. Our goal was to justify MMEF% predicted as a surrogate of LCI for assessment of bronchiectasis.

## Methods

### Patients

Between March 2014 and September 2015, consecutive bronchiectasis patients aged 18 years or greater were enrolled from First Affiliated Hospital of Guangzhou Medical University. Bronchiectasis was confirmed by chest HRCT (effective within 12 months), provided the internal diameter of bronchi exceeded that of accompanying pulmonary artery, or a lack of normal tapering in bronchial internal diameter, or bronchi being visible within 1 cm to the pleura. All patients remained exacerbation-free for at least 4 weeks. Exacerbation denoted at least 3 criteria lasting for 24 hrs or longer: significantly increased cough frequency; increased sputum purulence or volume; dyspnea; chest pain; T > 38 °C; exercise intolerance; hemoptysis; increased pulmonary infiltration^15^,^16^,^17^. We excluded patients with malignancy, upper respiratory tract infection or antibiotics use within 4 weeks, and those who could not cooperate with the measurements.

Ethics Committee of First Affiliated Hospital of Guangzhou Medical University gave ethics approval. Written informed consent was obtained from all patients.

### Study design

Study 1 was a cross-sectional investigation exploring the concordance of LCI and MMEF% predicted, and their association with clinical parameters in clinically stable bronchiectasis. Patients underwent history inquiry, multiple-breath nitrogen wash-out test, spirometry, and sputum culture.

To enable the comparison of the dynamic changes in LCI and MMEF% predicted, patients who experienced acute exacerbation were invited to participate in exacerbation and post-antibiotic treatment reassessments (Study 2). For the exacerbation visit, multiple-breath nitrogen wash-out test and spirometry were performed prior to treatment. At 1 week after completion of 14-day antibiotic therapy^18^, patients were invited for participation in post-antibiotic treatment visit. This enabled the comparison of changes in LCI and MMEF during exacerbation and post-antibiotic treatment visit.

The study was conducted in accordance with the STROBE guideline.

### HRCT scores

Chest HRCT within 12 months was evaluated by a radiologist blinded to patient’s allocation. HRCT score was determined on lobar basis (lingular treated as a separate lobe). Bronchiectasis was scaled using modified Reiff score (maximum: 18)^18^,^19^,^20^. Other features (heterogeneity, cystic bronchiectasis, unilateral/bilateral bronchiectasis) were also evaluated.

### Spirometry

We performed spirometry using spirometers (QUARK PFT, COSMED Inc., Italy) based on international guidelines^21^. Results were derived from 3 technically repeatable maneuvers, with between-maneuver variation <5% or 200 ml in forced vital capacity (FVC) and FEV_1_. Maximal FVC and FEV_1_ were reported. MMEF was selected from the best maneuver, that is, the maneuver with the largest sum of FVC and FEV_1_. Predicted values were derived from the models proposed by Zheng *et al*.^22^.

### Measurement of LCI

LCI was measured prior to spirometry, with the multiple-breath nitrogen wash-out technique^23^,^24^, by using the validated QUARK PFT real-time gas-analyzer (COSMED Inc., Italy) which has been employed for our routine clinical practice. The instrument has been calibrated to ensure the assay accuracy each day, prior to the measurement. The accuracy of the gas analyzer measuring nitrogen concentration was approximately 1% at start-of-test and 0.2% at end-of-test.

Patients were seated with a nose clip applied, and breathed in pure oxygen gas from the closed circuit through the mouthpiece whilst avoiding gas leakage. Patients were requested to maintain a steady respiratory rate of 12–16 breaths per minute, with the tidal volume of approximately 1.0 L (which can be graphically displayed on the computer screen, in a real-time fashion). Artifacts, such as cough, breathe with irregular small volumes, evidence of significantly trapped gas with larger breaths, or glottis closure, should also be avoided throughout the measurement. The proper maneuvers were repeated until the exhaled nitrogen concentration reached to 1/40^th^ of the original concentration (typically 2.5%) or lower, or the test exceeded the maximal allowable duration (typically 7.0 minutes, at least 6 lung turnovers included). At least two measurements with 10-minute intervals (which exceeded the single wash-out time to allow for nitrogen concentration to return to baseline levels) were performed^23^, which enabled the calculation of mean LCI. We discarded any maneuver in case the difference in functional residual capacity was 15% or greater, evidence of gas leakage or irregular breathing^23^,^24^,^25^.

LCI denoted the number of lung volume turnovers (cumulative expired volume divided by functional residual capacity) which enabled the reduction in end-tidal nitrogen concentration to 1/40^th^ of its initial concentration^11^. Higher LCI denoted a greater magnitude of ventilation heterogeneity.

### Sputum bacteriology

Sputum was collected at each assessment time. Fresh sputum was sampled during hospital visits. Following removal of oral cavity debris, patients expectorated into sterile container for culture, within 2 hours of sampling. Hypertonic saline (3% ~ 5%) induction was applied as appropriate^26^.

*Pseudomonas aeruginosa* colonization denoted sputum culture positive for 2 or more occasions (at least 3 months apart) within the nearest one year.

See details in online supplement.

### Disease severity assessment

*Bronchiectasis Severity Index* (BSI) was used to evaluate disease severity. The BSI was an integrated metric consisting of the age, body-mass index, prior exacerbation and prior hospitalization in the preceding year, Medical Research Council dyspnea score, FEV_1_ predicted%, P. *aeruginosa* colonization, colonization with other PPMs and the number of bronchiectatic lobes, The established cut-off value of 0–4, 5–8, and 9 or greater corresponded to mild, moderate and severe bronchiectasis, respectively^27^,^28^.

Additionally, we utilized modified Reiff score for assessment of radiologic severity of bronchiectasis^15^. The HRCT score was assessed on a lobar basis (lingular lobe as a separate lobe). For individual lobes, the extent of bronchiectasis was scored (0 for no, 1 for tubular, 2 for varicose and 3 for cystic bronchiectasis). Total HRCT score was derived by summing the score of 6 lung lobes (maximal total score: 18). We classified the HRCT score into tertiles: 0–6 for mild bronchiectasis, 7–12 for moderate bronchiectasis, and 13–18 for severe bronchiectasis^15^.

### Statistical analysis

SPSS 16.0 package (SPSS Inc., Chicago, USA) and Graphpad Prism 5.0 (Graphpad Inc., San Diego, USA) were employed for statistical analyses.

Numeric data were expressed as mean ± standard deviation or otherwise median (interquartile range) and compared with independent t-test or Mann-Whitney test, whilst categorical data were presented as No. (%) and compared using chi-square tests.

Receiver operation characteristic curve was constructed to compare LCI or MMEF% predicted in discriminating moderate-to-severe or severe bronchiectasis, along with area under curve (AUC), 95% confidence intervals (95% CI), sensitivity, specificity, accuracy and cut-off levels. We used kappa statistic to calculate agreement (and 95% CI) for dichotomous results of LCI and MMEF% predicted (lower and upper 50^th^ percentile), correcting for chance. Correlation between LCI and MMEF% predicted in different subgroups was analyzed with partial correlation model, adjusting for age, sex, body-mass index and baseline FEV_1_% predicted.

We utilized generalized linear mixed model to determine clinical variable attributes’ impacts on LCI and MMEF% predicted. Fixed-effect estimates were calculated with sex, sputum bacteriology (*Pseudomonas aeruginosa* or non-*Pseudomonas aeruginosa* colonized), cystic bronchiectasis (yes or no), heterogeneity (yes or no) and bilateral bronchiectasis (yes or no) as factors, and the number of bronchiectatic lobes, HRCT total score, age and baseline FEV_1_% predicted as covariates.

Paired t-test or Mann-Whitney test was employed to compare changes in LCI or MMEF% predicted at exacerbation and post-antibiotic treatment visit.

We have also performed sensitivity analyses in moderate-to-severe bronchiectasis, defined with the BSI (BSI <5, 5 ≤ BSI < 9, and BSI ≥ 9) and chest HRCT score (HRCT score <7, 7 ≤ HRCT score < 13, and HRCT score ≥13)^15^,^27^, respectively. The BSI evaluates disease severity in terms of future risks of exacerbation, hospitalization, and mortality, whereas HRCT score primarily reflects radiologic severity of bronchiectasis. Because the discordant BSI score and HRCT score has been observed in previous studies^15^,^17^, we sought to validate our findings according to composite disease severity and radiologic severity.

*P* < 0.05 was deemed statistically significant for all analyses.

## Results

### Patient recruitment

**Figure 1:**
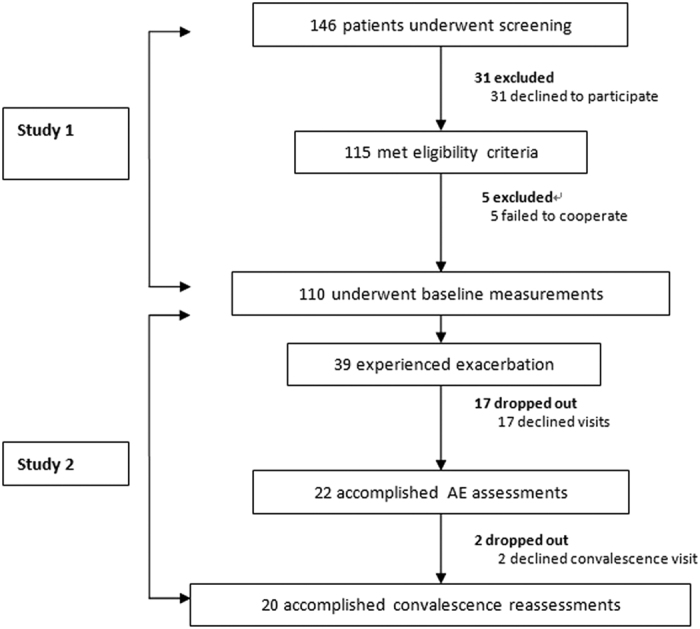
Patient recruitment flowchart Of 146 patients who underwent screening, 115 successfully participated in baseline measurements. Of these patients, 39 experienced Exacerbations and 22 accomplished exacerbation visits.

Of 146 patients who underwent screening, 115 were included in study 1. Of 39 patients who experienced exacerbation during the study, 22 participated in study 2 (Fig. 1).

### Characteristics of bronchiectasis patients

The clinically stable cohort consisted mainly of middle-aged females (mean: 44.6 yrs) who were mostly never-smokers (91.8%). Median HRCT score and BSI was 8.0 and 6.0, respectively. *Pseudomonas aeruginosa* was most commonly isolated (35.5%) from sputum, followed by normal flora (32.7%). Mucolytics were commonly used concomitant medications. The predominant underlying causes were idiopathic and post-infectious. None of the cases was deemed to be related to chronic obstructive pulmonary disease. Other concomitant diseases included hypertension (n = 4), psychiatrist-diagnosed depression (n = 1) and multiple hepatic cysts (n = 1). Four patients had a family history of bronchiectasis.

The exacerbation cohort did not differ statistically in all these parameters. (Table 1).

### Baseline LCI and MMEF

The mean baseline LCI and MMEF% predicted was 15.3 and 47.2%, respectively. For two acceptable measurements, the mean coefficient of variation was 4.37% and 2.78% for LCI and MMEF% predicted, respectively.

All 110 bronchiectasis patients had increased LCI (>7.5) and 79 patients had MMEF% predicted <65%. Baseline median was 14.70 and 45.3% for LCI and MMEF% predicted, respectively. Forty-four patients each (40%) had LCI <14.70 and MMEF% predicted >45.3%, and LCI ≥14.70 and MMEF% predicted ≤45.3%. Eleven patients each (10%) had LCI <14.70 and MMEF% predicted ≤45.3%, and LCI ≥14.70 and MMEF% predicted >45.3%.

### LCI and MMEF for discriminating moderate-to-severe bronchiectasis

**Figure 2:**
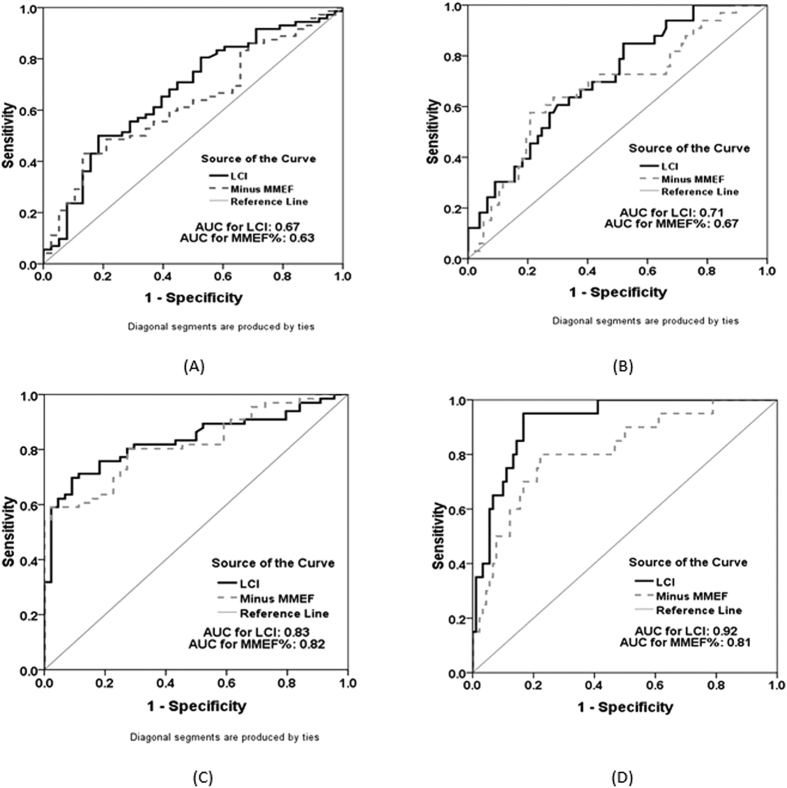
Diagnostic performance of LCI and MMEF predicted% in clinically stable bronchiectasis. (**A**), Diagnostic performance of LCI and MMEF% predicted in patients with Bronchiectasis Severity Index of 5 or greater; AUC: 0.672, 95% CI: (0.57, 0.78) for LCI; AUC: 0.63, 95% CI: (0.52, 0.74) for MMEF% predicted. (**B**), Diagnostic performance of LCI and MMEF% predicted in patients with Bronchiectasis Severity Index of 9 or greater; AUC: 0.71, 95% CI: (0.60, 0.81) for LCI; AUC: 0.67, 95% CI: (0.56, 0.78) for MMEF% predicted. (**C**), Diagnostic performance of LCI and MMEF% predicted in bronchiectasis patients with HRCT score of 7 or greater; AUC: 0.83, 95% CI: (0.76, 0.91) for LCI; AUC: 0.82, 95% CI: (0.75, 0.90) for MMEF% predicted (**D**), Diagnostic performance of LCI and MMEF% predicted in bronchiectasis patients with HRCT score of 13 or greater. AUC: 0.92, 95% CI: (0.87, 0.98) for LCI; AUC: 0.81, 95% CI: (0.71, 0.92) for MMEF% predicted AUC: area under curve The bold black line indicated the LCI, whereas the grey dotted line represented the minus MMEF% predicted (for direct comparison purposes).

Overall, LCI and MMEF% predicted had similar power in discriminating moderate-to-severe bronchiectasis from mild bronchiectasis (Fig. 2, Table S1). The diagnostic value of LCI and MMEF% predicted was higher for discriminating severe bronchiectasis alone (AUC: 0.71 for LCI; AUC: 0.67 for MMEF% predicted) compared with moderate-to-severe bronchiectasis (AUC: 0.67 for LCI; AUC: 0.63 for MMEF% predicted).

Similarly, the diagnostic value of LCI and MMEF% predicted was higher for discriminating patients with HRCT score of 13 or greater (AUC: 0.92 for LCI; AUC: 0.81 for MMEF% predicted) than those who had HRCT score of 7 or greater (AUC: 0.83 for LCI; AUC: 0.82 for MMEF% predicted).

### Diagnostic value of LCI and MMEF to discriminate patients with or without FEV_1_ >80% predicted

As shown in Table S2, MMEF% predicted conferred higher assay specificity (0.97 vs. 0.80) yet an identical sensitivity (0.79 vs. 0.79) compared with LCI. Hence, MMEF% predicted would not be inferior to LCI in identifying bronchiectasis with relatively preserved lung function.

### Differences in LCI and MMEF according to disease severity

**Figure 3:**
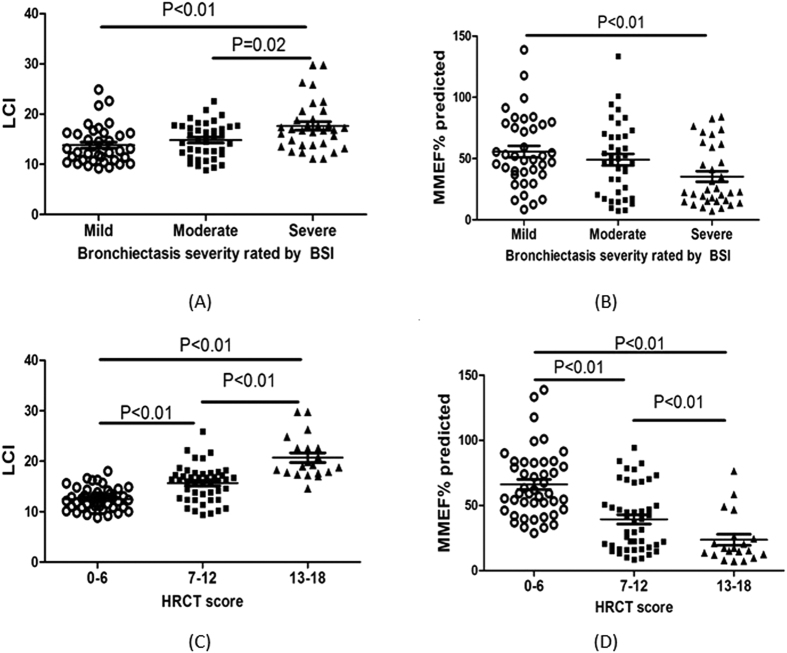
Association between LCI and MMEF% predicted and disease severity. (**A**), Association between LCI and the Bronchiectasis Severity Index; Thirty-eight patients had a Bronchiectasis Severity Index of 4 or lower, 39 patients had a Bronchiectasis Severity Index of 5 or greater and 8 or lower, and 33 patients had a Bronchiectasis Severity Index of 9 or greater. (**B**), Association between MMEF% predicted and the Bronchiectasis Severity Index; Thirty-eight patients had a Bronchiectasis Severity Index of 4 or lower, 39 patients had a Bronchiectasis Severity Index of 5 or greater and 8 or lower, and 33 patients had a Bronchiectasis Severity Index of 9 or greater. (**C**), Association between LCI and the HRCT total score; Forty-four patients had an HRCT total score of 6 or lower, 46 patients had an HRCT total score of 7 or greater and 12 or lower, and 20 patients had an HRCT total score of 13 or greater. (**D**), Association between MMEF% predicted and the HRCT total score. Forty-four patients had an HRCT total score of 6 or lower, 46 patients had an HRCT total score of 7 or greater and 12 or lower, and 20 patients had an HRCT total score of 13 or greater.

Both LCI and MMEF predicted% varied considerably among different levels of disease severity. Patients with greater severity of bronchiectasis (BSI ≥9 or HRCT score ≥13) had significantly higher levels of LCI and lower MMEF predicted% than their counterparts (both P < 0.01). Patients with BSI <5 or HRCT score <7 consistently presented with significantly lower levels of LCI and higher MMEF predicted% than their counterparts (both P < 0.01). (Fig. 3).

### Correlation between LCI and MMEF in clinically stable bronchiectasis

Following adjustment with age, sex and body-mass index, the LCI correlated negatively with MMEF% predicted (r = −0.64, P < 0.01), which was independent of the disease severity as rated by the *BSI* (0–4 points: r = −0.61, P < 0.01; 5–8 points: r = −0.64, P < 0.01; 9 points or greater: r = −0.65, P < 0.01) and HRCT total score (0–6 points: r =  −0.48, P < 0.01; 7–12 points: r = −0.71, P < 0.01) except for those with HRCT score of 13–18 (r = −0.12, P = 0.66). (Table 2).

However, when further adjusted with FEV_1_% predicted, the significant correlation existed only in patients with moderate bronchiectasis (r =  −0.49, P < 0.01) and HRCT score of 7 or greater and 12 or lower (r =  −0.43, P < 0.01) (Table S3).

No significant correlation between LCI and other spirometric parameters such as FEV_1_/FVC% could be found (Table S4).

### Concordance of LCI and MMEF in reflecting different clinical characteristics of bronchiectasis

Overall, compared with MMEF% predicted, higher levels of LCI had consistently greater concordance to reflecting 3 or more bronchiectatic lobes [κ = 0.527, 95% CI: (0.376, 0.678)], HRCT total score of 9 or higher [κ = 0.527, 95% CI: (0.376, 0.678)], BSI of 5 or higher [κ = 0.309, 95% CI: (0.133, 0.485)], cystic bronchiectasis [κ = 0.291, 95% CI: (0.126, 0.456)] and *Pseudomonas aeruginosa* colonization [κ = 0.200, 95% CI: (0.035, 0.365)]. Both variables had equal capacity in identifying patients with bilateral bronchiectasis as opposed to unilateral bronchiectasis [κ = 0.291, 95% CI: (0.154, 0.423)]. Not surprisingly, lower MMEF% predicted had greater capacity in identifying patients with FEV_1_ 80% predicted or lower [κ = −0.364, 95% CI: (−0.519, −0.209)]. However, none of these differences reached statistical significance because 95% CI for both variables was overlapped. (Table 3).

Subgroup analyses when stratifying the BSI (BSI <5, 5 ≤ BSI < 9, and BSI ≥ 9) or HRCT total score (HRCT score <7, 7 ≤ HRCT score < 13, and HRCT score ≥13) showed slightly variable findings, but consistently demonstrated that LCI and MMEF% predicted had comparable discriminatory capacity in different disease severity categories (Tables S5–S10).

### Clinical variable attributes’ impacts on LCI and MMEF

Both LCI and MMEF% predicted were significantly correlated with the number of bronchiectatic lobes, HRCT total score, age, FEV_1_% predicted, *Pseudomonas aeruginosa* colonization, and the presence of cystic bronchiectasis, ventilation heterogeneity, and bilateral bronchiectasis (all P < 0.001). The overall effect sizes were numerically greater for MMEF% predicted than for LCI. MMEF% predicted, but not LCI, correlated significantly with sex. (Table 4).

Despite slightly variable results, subgroup analyses which stratified the BSI (BSI <5, 5 ≤ BSI < 9, and BSI ≥ 9) or HRCT total score (HRCT score <7, 7 ≤ HRCT score < 13, and HRCT score ≥13) showed comparable clinical variable attributes’ impacts on LCI and MMEF% predicted (Tables S11–S16).

### Changes in LCI and MMEF during exacerbation and post-antibiotic treatment visit

**Figure 4:**
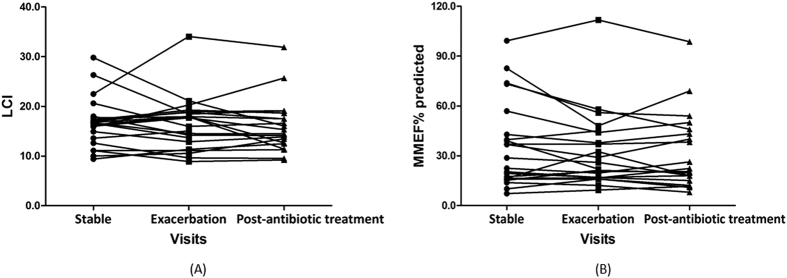
Changes in LCI and MMEF% predicted from baseline to acute exacerbation and post-antibiotic treatment visit (**A**). Changes in LCI predicted from baseline to acute exacerbation and post-antibiotic treatment visit in all bronchiectasis patients; (**B**). Changes in MMEF% predicted from baseline to acute exacerbation and post-antibiotic treatment visit in all bronchiectasis patients.

Finally, we compared the variation in LCI and MMEF% predicted during exacerbation and post-antibiotic treatment visit (Table 5, Fig. 4). The overall changes were small (within 5% of baseline levels) and did not reach statistical significance. Subgroup analyses showed that the above finding was also applied, regardless of the subgroup when stratifying the BSI (BSI <5, 5 ≤ BSI < 9, and BSI ≥ 9) or HRCT total score (HRCT score <7, 7 ≤ HRCT score < 13, and HRCT score ≥13).

## Discussion

### Principal findings

Whilst FEV_1_ primarily associates with large-airway lesions, LCI sensitively reflects ventilation heterogeneity which correlated with disease severity and distal airway inflammation^2^,^3^,^4^. Because small airway disorder contributes to ventilation heterogeneity, we specifically focused on comparing the diagnostic value of LCI and MMEF and their correlation with clinical parameters. LCI demonstrated similar diagnostic value compared with MMEF in bronchiectasis; both parameters correlated independently with FEV_1_ and had similarly limited usefulness in identifying onset of exacerbation.

### Critiques and interpretation

Currently, data directly comparing LCI and MMEF in bronchiectasis have been scarce. Our findings were discordant with those reported by Rowan *et al*.^4^, who found that LCI yielded significantly higher diagnostic value [AUC: 0.96, 95% CI: (0.93, 0.99)] than MMEF [AUC: 0.76, 95% CI: (0.67, 0.86)], although these parameters had similar magnitudes of correlation with HRCT parameters, including percent bronchiectasis, airway thickening, mucus plugging, parenchymal and air trapping. The observed disparity might be partially attributable to baseline disease severity–most patients had mild-to-moderate airflow obstruction (mean FEV_1_: 76.5% predicted) in Rowan *et al*.’s study^4^, whereas mean FEV_1_ predicted was 61.5% herein. Despite the lack of assessment of MMEF, Gustafsson *et al*.^11^ did show that FEF_25%_ (another small-airway spirometric parameter) and LCI had consistently greater concordance, and assay sensitivity and specificity than FEV_1_ in reflecting the presence of bronchiectasis, HRCT score and air trapping in patients with cystic fibrosis. Nonetheless, our findings were further validated in severe bronchiectasis, suggesting that MMEF% predicted might be as valuable as LCI in discriminating moderate-to-severe bronchiectasis, as evidenced by their comparable sensitivity, specificity and concordance of reflecting clinical characteristics.

Our findings mirrored those by Irving *et al*.^9^, showing that LCI correlated negatively with MMEF% predicted not only in primary ciliary dyskinesia and cystic fibrosis, but also in adults with bronchiectasis. Following adjustment with FEV_1_ predicted%, the significant correlation between LCI and MMEF% predicted remained in moderate bronchiectasis only, indicating that MMEF was partially dependent of FEV_1_. Notably, LCI was dependent on age, HRCT total score and FEV_1_, which partially agreed with the recent literature^29^ which indicated the age dependence of LCI. Because MMEF is a mixture of large and small airway gas-flow, the correlation with demographic and clinical parameters might have been considerably tempered. Notably, both parameters correlated significantly with HRCT characteristics, including cystic bronchiectasis, heterogeneity and the number of bronchiectatic lobes. Therefore, LCI and MMEF% predicted are not only physiologic measures, but also have profound implications in reflecting lung structural damage. Theoretically, small airway lesion preceded the development of clinically apparent bronchiectasis, rendering early-stage airway impairment to readily translate into clinically significant reduction in MMEF and/or increase in LCI. The subgroup analyses in our study have justified the usefulness of both parameters in reflecting physiologic and structural impairment in milder forms of bronchiectasis.

Intriguingly, no notable differences were observed in LCI or MMEF during exacerbation and post-antibiotic treatment visit. Our pilot studies^15^,^30^ have verified that, during exacerbation, lung function parameters including spirometric small-airway indices and impulse oscillometry parameters changed insignificantly, except for FVC and FEV_1_ (within 5% baseline levels). Our study further added that LCI, the parameter closely associated with ventilation heterogeneity, actually changed little during exacerbation. This has again challenged our hypothesis that worsened ventilation heterogeneity characterized the exacerbation. This was supported by Grillo *et al*.^31^, who documented that LCI was unresponsive to two short-term courses of chest physiotherapy plus antibiotics therapy for exacerbation. Similarly, a meta-analysis of cystic fibrosis showed that LCI decreased by 0.4 units or 2.5% following antibiotics treatment for exacerbation^32^, which was statistically significant but clinically negligible. Our findings reaffirmed that MMEF changed insignificantly during exacerbation and post-antibiotic treatment visit compared with baseline levels. In view of contradictory findings with research hypothesis, the pathophysiology of exacerbation should be re-visited.

### Strengths and limitation

We have comprehensively compared LCI and MMEF on their diagnostic value, concordance and correlation with clinical parameters with fixed-effect models, dynamic comparison from stability to post-antibiotic treatment visit, coupled with the sensitivity analyses.

Nonetheless, major limitations are:

1. Our findings might have been biased by monocentric study design. However, our cohort still represented characteristics of local bronchiectasis population^33^.
2. We did not include longitudinal follow-up visits because some patients have been recruited for less than one year, therefore prognostic significance of LCI and MMEF remains unclear.
3. It would be interesting to unravel the diagnostic performance of LCI and MMEF% predicted in mild bronchiectasis; however, we were unable to comment on this because healthy subjects have not been recruited for comparison in this study.
4. LCI alone could not discriminate conductive or acinar ventilation heterogeneity. The sample size in certain subgroups (i.e. HRCT score ≥13) was relatively small, hence our sample size might not be sufficient to power sensitivity analyses.
5. Although the use of MMEF has been criticized for the failure to contribute to clinical decision-making^34^, we found that MMEF correlated significantly with various clinical variables and that the coefficient of variation for two acceptable maneuvers was small. Furthermore, the usefulness of MMEF in terms of correlation with airway hyperresponsiveness and inflammation in asthma^35^,^36^ and asthma-like symptoms^37^ has been demonstrated. Despite different settings, it is still likely that MMEF% predicted would have clinical significance to guide severity assessment of bronchiectasis.
6. Furthermore, raw LCI could be influenced by age, sex and FEV_1_% predicted, whilst our correlation analysis has fully adjusted for these covariates. The unmeasured factors might have also affected the diagnostic performance of LCI and MMEF. We only adopted a single gas to determine LCI.
7. Finally, pulmonary imaging (i.e. polarized gases) is needed to better elucidate the observed differences in diagnostic performance of LCI and MMEF.

### Clinical significance

Conventional lung function tests evaluate, if any, the magnitude of impairment in lung volume, airway obstruction and lung diffusing capacity. However, small airway disorders frequently precede large airway disorders. The degree of ventilation heterogeneity can reflect the magnitude of small airway disorder which correlates with patient’s overall well-being. Therefore, measurement of ventilation heterogeneity with LCI (i.e. using multiple-breath inert gas washout techniques) may have complementary roles for assessment of the severity in debilitating diseases such as bronchiectasis.

Despite the high assay sensitivity and specificity of LCI, the lack of cooperation, the longer duration of measurement and the requirement for gas source (i.e. pure oxygen, sulfur hexafluoride) and equipment might have collectively constrained its clinical usefulness for evaluating lung function in bronchiectasis. Hence, although conventional measures (such as FEV_1_) were inferior to LCI in terms of diagnostic performance (which has been corroborated by our findings, as shown in Figure E1), MMEF which reflects small-airway disorder may be a surrogate endpoint, particularly for bronchiectasis patients who could not cooperate with the measurement or institutions without sophisticated testing instruments.

In light of the greater concordance and more significant correlation with clinical variables, LCI should be the endpoint with priority for assessing ventilation heterogeneity. However, because of the greater assay specificity of MMEF% predicted in discriminating bronchiectasis patients who have FEV_1_ >80% predicted, it is likely that LCI and MMEF% predicted would be complementary for assessment of lung structural changes in bronchiectasis patients with preserved lung function.

Importantly, neither LCI nor MMEF should be solely rested on to determine the severity of bronchiectasis exacerbation or confirm the recovery from exacerbation^38^.

### Clinical trial registry

Clinicaltrials.gov; No.: NCT01761214; URL: www.clinicaltrials.gov. 

## Additional Information

**How to cite this article**: Guan, W.- J. *et al*. Maximal mid-expiratory flow is a surrogate marker of lung clearance index for assessment of adults with bronchiectasis. *Sci. Rep.* **6**, 28467; doi: 10.1038/srep28467 (2016).

## Supplementary Material

Supplementary Information

## Figures and Tables

**Table 1:** Characteristics of bronchiectasis patients.

Parameter	Study 1 (n = 110)	Study 2 (n = 22)*
Anthropometry		
Age (years)	44.6 ± 14.1	43.1 ± 14.0
Females (No., %)	67 (60.9%)	14 (63.6%)
BMI (kg/m^2^)	20.1 (5.2)	19.2 ± 2.6
Never-smokers (No., %)	101 (91.8%)	21 (95.5%)
History		
Duration of symptoms (yrs)	12.0 (14.0)	12.5 (9.2)
No. of exacerbations within 2 yrs	3.0 (3.0)	3.0 (3.0)
HRCT findings		
No. of bronchiectatic lobes	4.0 (4.0)	4.5 (2.2)
HRCT total score	8.0 (7.0)	8.0 (4.2)
Disease severity		
*Bronchiectasis Severity Index*	6.0 (5.0)	7.5 (7.3)
Sputum bacteriology when clinically stable		
*Pseudomonas aeruginosa* (No., %)	39 (35.5%)	9 (40.9%)
*Haemophilus influenzae* (No., %)	10 (9.9%)	2 (9.9%)
Other pathogenic bacteria (No., %)**	25 (22.7%)	4 (18.2%)
Commensals (No., %)	36 (32.7%)	7 (31.8%)
Medications ever used within 6 months#		
Inhaled corticosteroids (No., %)	26 (23.6%)	3 (13.6%)
Mucolytics (No., %)	83 (75.5%)	19 (86.4%)
Macrolides (No., %)	41 (37.3%)	6 (27.3%)
Underlying causes##		
Post-infectious (No., %)	34 (30.9%)	8 (36.4%)
Immunodeficiency (No., %)	17 (15.5%)	2 (9.9%)
Miscellaneous known findings (No., %)	16 (14.5%)	3 (13.6%)
Idiopathic (No., %)	47 (42.7%)	10 (45.5%)

Numerical data were presented as either mean ± standard deviation (SD) or median (interquartile range, IQR) as appropriate. NA: not applicable.

None of the patients was regularly using inhaled, oral or systemic antibiotics.

^*^The number of patient-reported exacerbation events that did not meet our criteria was not recorded and therefore we could not perform sensitivity analysis on this subgroup.

^**^Other pathogenic bacteria for the clinically stable cohort included *Haemophilus parainfluenzae* (n** = **8, 7.3%), *Escherichia coli* (n** = **5, 4.5%), *Klebsiella pneumoniae* (n** = **4, 3.6%), *Serratia marcescens* (n** = **2, 1.8%), *Streptococcus pneumoniae* (n** = **1, 0.9%), *Moraxella catarrhalis* (n** = **1, 0.9%), *Achromobacter xylosoxidans* (n** = **1, 0.9%), *Proteus mirabilis* (n** = **1, 0.9%), *Haemophilus haemolyticus* (n** = **1, 0.9%) and *Bordetella bronchiseptica* (n** = **1, 0.9%). The underlying causes of bronchiectasis were determined after meticulous testing recommended by *British Thoracic Society* guidelines and group discussion (W.J.G., J.J.Y. and Y.H.G.). Further details will be published elsewhere.

^#^Most patients had ever used more than one category of medications within the last 6 months.

^##^Dual underlying causes were determined in a minority of patients, thus the cumulative percentage was greater than 100%. Miscellaneous causes consisted of allergic bronchopulmonary aspergillosis, gastroesophageal reflux disease, asthma, diffuse panbronchiolitis, Kartagener syndrome, non-tuberculous mycobacteria disease, Young’s syndrome, rheumatoid arthritis, lung sequestration syndrome, and lung malformation.

**Table 2:** Comparison and correlation of LCI and MMEF% predicted in different subgroups.

	No.	LCI Median (95% CI)	MMEF predicted% Median (95% CI)	r Value of Correlation#	P Value of Correlation#
All patients	110	14.70 (14.49, 16.13)	45.30 (41.72, 52.72)	−0.64	<0.01
0≤ BSI ≤4	38	12.90 (12.57, 15.05)	50.64 (46.06, 65.23)	−0.61	<0.01
5≤ BSI ≤8	39	14.60 (13.68, 15.96)	47.02 (39.43, 58.72)	−0.64	<0.01
BSI ≥9	33	16.90 (15.89, 19.37)	22.87 (26.55, 44.09)	−0.65	<0.01
P value*	–	<0.01	<0.01	–	–
HRCT score ≤6	44	12.40 (11.84, 13.18)	66.14 (58.26, 74.01)	−0.48	<0.01
7≤ HRCT score ≤12	46	16.20 (14.60, 16.69)	34.26 (32.11, 46.61)	−0.71	<0.01
HRCT score ≥13	20	19.15 (18.73, 22.69)	16.32 (14.78, 32.57)	−0.12	0.66
P value**	–	<0.01	<0.01	–	–

95% CI: 95% confidence interval LCI: lung clearance index; MMEF: maximal mid-expiratory flow.

^*^Comparison among patients with BSI less than 5, BSI of 5 or greater and 8 or lower, and those with BSI of 9 or greater.

^**^Comparison among patients with HRCT total score less than 7, HRCT total score of 7 or greater and 12 or lower, and those with HRCT total score of 13 or greater.

^#^Because LCI and MMEF% predicted might have been biased by age, sex and body-mass index, the correlation between LCI and MMEF% predicted in different subgroups was analyzed with partial correlation model, adjusting for the patient’s age, sex and body-mass index.

**Table 3:** Concordance of lung clearance index and maximal mid-expiratory flow with clinical variables in stable bronchiectasis.

	Lung clearance index				Maximal mid-expiratory flow			
	Lower 50^th^ percentile*	Upper 50^th^ percentile*	Total	Concordance (95% CI)	Lower 50^th^ percentile**	Upper 50^th^ percentile**	Total	Concordance (95% CI)
≤3 bronchiectatic lobes	35	6	41	0.527 (0.376, 0.678)	33	8	41	0.455 (0.294, 0.616)
>3 bronchiectatic lobes	20	49	69		22	47	69	
Total	55	55	110	–	55	55	110	–
HRCT total score ≤9	51	22	73	0.527 (0.376, 0.678)	48	25	73	0.418 (0.257, 0.579)
HRCT total score >9	4	33	37		7	30	37	
Total	55	55	110	–	55	55	110	
BSI ≤5	33	16	49	0.309 (0.133, 0.485)	32	17	50	0.273 (0.095, 0.451)
BSI >5	22	39	61		23	38	60	
Total	55	55	110	–	55	55	110	–
Unilateral bronchiectasis	18	2	20	0.291 (0.154, 0.423)	18	2	20	0.291 (0.154, 0.423)
Bilateral bronchiectasis	37	53	90		37	53	90	
Total	55	55	110	–	55	55	110	–
Tubular/varicose bronchiectasis	25	9	34	0.291 (0.126, 0.456)	23	11	34	0.218 (0.049, 0.387)
Cystic bronchiectasis	30	46	76		32	44	76	
Total	55	55	110	–	55	55	110	–
Homogeneity	25	6	31	0.345 (0.186, 0.504)	23	8	31	0.273 (0.110, 0.436)
Heterogeneity	30	49	79		32	47	79	
Total	55	55	110	–	55	55	110	–
No *Pseudomonas aeruginosa* colonized	45	34	79	0.200 (0.035, 0.365)	44	35	79	0.164 (−0.003, 0.331)
*Pseudomonas aeruginosa* colonized	10	21	31		11	20	31	
Total	55	55	110	–	55	55	110	–
FEV_1_ predicted ≤80%	5	25	30	−0.364 (−0.519, −0.209)	29	1	30	0.509 (0.366, 0.652)
FEV_1_ predicted >80%	50	30	80		26	54	80	
Total	55	55	110	–	55	55	110	–

Data are presented with counts unless otherwise stated. 95% CI: 95% confidence interval LCI: lung clearance index; MMEF: maximal mid−expiratory flow.

^*^For LCI, “Low” denoted the values being equal to or lower than the median (14.70), whereas “high” indicated the values being higher than the median (14.70).

^**^For MMEF predicted%, “Low” denoted the values being equal to or lower than the median (45.3%), whereas “high” indicated the values being higher than the median (45.3%).

**Table 4:** Fixed-effect estimates in multivariate linear mixed model of the clinical variable attributes’ impacts on lung clearance index and maximal mid-expiratory flow.

	LCI			MMEF predicted%		
	Estimate	P value	95% CI	Estimate	P value	95% CI
Intercept	**15**.**571**	**<0**.**001**	**14**.**115**, **17**.**028**	**−19**.**434**	**<0**.**001**	**−20**.**890**, **−17**.**978**
No. of bronchiectatic lobes	**−0**.**254**	**0**.**046**	**−0**.**505**, **−0**.**004**	**−1**.**519**	**<0**.**001**	**−1**.**769**, **−1**.**269**
HRCT total score*	**0**.**477**	**<0**.**001**	**0**.**372**, **0**.**582**	**0**.**521**	**<0**.**001**	**0**.**416**, **0**.**626**
Age	**0**.**051**	**<0**.**001**	**0**.**037**, **0**.**066**	**0**.**028**	**<0**.**001**	**0**.**014**, **0**.**043**
FEV_1_ predicted%	**−0**.**080**	**<0**.**001**	**−0**.**091**, **−0**.**068**	**1**.**048**	**<0**.**001**	**1**.**037**, **1**.**060**
Sex	–	–	–	–	–	–
Males	Reference	Reference	Reference	Reference	Reference	Reference
Females	0.371	0.072	−0.034, 0.776	**1**.**542**	**<0**.**001**	**1**.**137**, **1**.**947**
Sputum bacteriology	–	–	–	–	–	–
*Pseudomonas aeruginosa* colonized	Reference	Reference	Reference	Reference	Reference	Reference
No *Pseudomonas aeruginosa* colonized	**−0**.**484**	**0**.**037**	**−0**.**939**, **−0**.**028**	**−2**.**576**	**<0**.**001**	**−3**.**032**, **−2**.**120**
Cystic bronchiectasis	–	–	–	–	–	–
Yes	Reference	Reference	Reference	Reference	Reference	Reference
No	**0**.**748**	**0**.**004**	**0**.**245**, **1**.**252**	**−3**.**082**	**<0**.**001**	**−3**.**586**, **−2**.**578**
Heterogeneity	–	–	–	–	–	–
Yes	Reference	Reference	Reference	Reference	Reference	Reference
No	**−0**.**518**	**0**.**038**	**−1**.**006**, **−0**.**029**	**1**.**087**	**<0**.**001**	**0**.**598**, **1**.**575**
Bilateral bronchiectasis	–	–	–	–	–	–
Yes	Reference	Reference	Reference	Reference	Reference	Reference
No	**−0**.**857**	**0**.**008**	**−1**.**494**, **−0**.**219**	**3**.**420**	**<0**.**001**	**2**.**783**, **4**.**058**

95% CI: 95% confidence interval LCI: lung clearance index; MMEF: maximal mid-expiratory flow Data in bold indicated the statistical analyses with significance.

^*^Modified Reiff score.

**Table 5:** Changes in LCI and MMEF% predicted during exacerbation and post-antibiotic treatment visit.

	Parameter	Baseline levels	Bronchiectasis exacerbation		Post-antibiotic treatment visit	
			Change from baseline Median (95% CI)	P value*	Change from exacerbation Median (95% CI)	P value**
All patients	No.	–	22	–	20	–
	LCI	16.65 (4.43)	0.38 (−2.34, 1.78)	0.50	−0.06 (−1.86, 0.72)	0.36
	MMEF predicted%	25.60 (30.05)	−2.00 (−8.23, 2.18)	0.28	−0.75 (−4.26, 3.69)	0.82
BSI <5	No.	–	7	–	6	–
	LCI	15.07 ± 2.63	0.39 (−1.68, 2.46)	0.66	−0.17 (−2.01, 1.66)	0.82
	MMEF predicted%	36.80 (22.50)	−0.16 (−10.62, 10.31)	0.97	4.72 (−5.35, 14.80)	0.28
5 ≤ BSI < 9	No.	–	6	–	5	–
	LCI	14.75 ± 3.11	−0.53 (−3.59, 2.53)	0.67	0.03 (−4.81, 4.86)	0.99
	MMEF predicted%	41.07 ± 22.17	4.09 (−4.82, 12.99)	0.29	−1.02 (−10.57, 8.53)	0.78
BSI ≥9	No.	–	9	–	9	–
	LCI	19.27 ± 6.30	0.04 (−5.53, 3.85)	0.68	0.20 (−3.42, 0.69)	.16
	MMEF predicted%	19.40 (36.20)	−2.40 (−15.45, 4.60)	0.43	−1.50 (−4.12, 8.03)	>.99
HRCT score <7	No.	–	8	–	6	–
	LCI	57.11 ± 25.53	1.50 (−0.09, 3.08)	0.06	−0.72 (−1.93, 0.50)	0.19
	MMEF predicted%	13.49 ± 2.84	9.38 (−2.96, 21.73)	0.12	−2.47 (−15.77, 10.83)	0.66
7 ≤ HRCT score < 13	No.	–	9	–	9	–
	LCI	19.40 (23.75)	−0.68 (−2.70, 1.35)	0.46	0.89 (−1.84, 3.61)	0.47
	MMEF predicted%	16.70 (1.55)	0.37 (−6.06, 6.80)	0.90	1.69 (−4.25, 7.63)	0.53
HRCT score ≥13	No.	–	5	–	5	–
	LCI	22.56 ± 5.61	1.62 (−10.60, 10.04)	>0.99	−1.33 (−4.38, 1.29)	0.31
	MMEF predicted%	14.82 ± 4.93	2.20 (−3.45, 5.89)	0.51	−1.50 (−4.02, 1.94)	0.39

95% CI: 95% confidence interval LCI: lung clearance index; MMEF: maximal mid-expiratory flow For baseline levels, data were expressed as mean ± standard deviation or otherwise median (interquartile range) if appropriate.

^*^P value of exacerbation level compared with that of baseline levels.

^**^P value of post-antibiotic treatment visit level compared with that of exacerbation levels.
