# Supplementary material for: Maximal mid-expiratory flow is a surrogate marker of lung clearance index for assessment of adults with bronchiectasis
Source: Sci Rep. 2016 Jun 24;6:28467. doi: 10.1038/srep28467 (PMC4919685; doi:10.1038/srep28467)
Supplement: Supplementary Information [file srep28467-s1.doc]

**Online supplement**

**Maximal mid-expiratory flow is a surrogate marker of lung clearance index for assessment of disease severity in bronchiectasis**

**Wei-jie Guan ^*1^, Ph.D., Jing-jing Yuan ^1^, M.Sc., Yong-hua Gao ^2^, Ph.D., Hui-min Li ^1^, M.T., Jin-ping Zheng ^1^, M.D., Rong-chang Chen ^1^, M.D., Nan-shan Zhong ^1^, M.D.**

^1^ State Key Laboratory of Respiratory Disease, National Clinical Research Center for Respiratory Disease, Guangzhou Institute of Respiratory Disease, First Affiliated Hospital of Guangzhou Medical University, Guangzhou, Guangdong, China

^2^ Department of Respiratory and Critical Care Medicine, First Affiliated Hospital of Zhengzhou University, Zhengzhou, Henan, China

**Corresponding author 1:** Nan-shan Zhong, M. D., State Key Laboratory of Respiratory Disease, National Clinical Research Center for Respiratory Disease, Guangzhou Institute of Respiratory Disease, First Affiliated Hospital of Guangzhou Medical University, Address: 151 Yanjiang Road, Guangzhou, Guangdong, China, Fax: +86-20-83062718, Phone: +86-20-83062718, E-mail: [nanshan@vip.163.com](mailto:nanshan@vip.163.com)

**Corresponding author 2:** Rong-chang Chen, M. D., State Key Laboratory of Respiratory Disease, National Clinical Research Center for Respiratory Disease, Guangzhou Institute of Respiratory Disease, First Affiliated Hospital of Guangzhou Medical University, Address: 151 Yanjiang Road, Guangzhou, Guangdong, China, Fax: +86-20-83062719, Phone: +86-20-83062719, E-mail: chenrc@vip.163.com

**E-mail address for all authors**

Dr. Wei-jie Guan: [battery203@163.com](mailto:battery203@163.com)

Ms Jing-jing Yuan: [jacyyjj@163.com](mailto:jacyyjj@163.com)

Dr. Yong-hua Gao: [gaoyonghuahust@163.com](mailto:gaoyonghuahust@163.com)

Ms Hui-min Li: [905488325@qq.com](mailto:905488325@qq.com)

Prof. Jin-ping Zheng: [jpzhenggy@163.com](mailto:jpzhenggy@163.com)

Prof. Rong-chang Chen: [chenrc@vip.163.com](mailto:chenrc@vip.163.com)

Prof. Nan-shan Zhong: [nanshan@vip.163.com](mailto:nanshan@vip.163.com)

**Author contributions:** W. J. G. and Y. H. G. drafted the manuscript; J. J. Y., J. P. Z., R. C. C. and N. S. Z. were responsible for patient recruitment; W. J. G., Y. H. G., H. M. L. and J. J. Y. collected individual data; W. J. G. performed statistical analyses; W. J. G., J. P. Z., R. C. C. and N. S. Z. contributed to study conception; R. C. C. and N. S. Z. provided critical review of the manuscript and approved the final submission.

**Methods**

**Sputum bacteriology**

Fresh sputum was sampled during hospital visits. Following removal of debris in oral cavity, patients expectorated into sterile container for bacterial culture. Hypertonic saline (3%~5%) induction, previously validated, was applied as appropriate. Sputum was sent for bacterial culture within 2 hours of sampling.

Blood and chocolate agar plates (Biomeurix, France) were adopted as culture media. Fresh sputum was homogenized with SPUTASOL (Oxoid SR089A, Cambridge, UK) and serially diluted with natural saline at concentrations of 10^-4^, 10^-5^ and 10^-6^. This was followed by addition of 10μl respective diluent to the plates with micropipette tube and inoculation using 10μl standardized rings. Plates were positioned in thermostatic box containing 5% carbon dioxide at 37℃ for overnight incubation.

*Pseudomonas aeruginosa* colonization denoted sputum culture positive for 2 or more occasions (at least 3 months apart) within 1 year.

**Measurement of LCI**

LCI was measured prior to spirometry, with the multiple-breath nitrogen wash-out technique, by using the validated QUARK PFT real-time gas-analyzer (COSMED Inc., Italy) which has been employed for our routine clinical practice. The instrument has been calibrated to ensure the assay accuracy each day, prior to the measurement. The accuracy of the gas analyzer measuring nitrogen concentration was approximately 1% at start-of-test and 0.2% at end-of-test.

Patients were seated with a nose clip applied, and breathed in pure oxygen gas from the closed circuit through the mouthpiece whilst avoiding gas leakage. Patients were requested to maintain a steady respiratory rate of 12-16 breaths per minute, with the tidal volume of approximately 1.0 L (which can be graphically displayed on the computer screen, in a real-time fashion). Artifacts, such as cough, breathe with irregular small volumes, evidence of significant trapped gas with larger breaths, or glottis closure, should also be avoided throughout the measurement. The proper maneuvers were repeated until the exhaled nitrogen concentration reached to 1/40^th^ of the original concentration (typically 2.5%) or lower, or the test exceeded the maximal allowable duration (typically 7.0 minutes, at least 6 lung turnovers included). At least two measurements with 10-minute intervals (which exceeded the single wash-out time to allow for nitrogen concentration to return to baseline levels) were performed, which enabled the calculation of mean LCI. We discarded any maneuver in case the difference in functional residual capacity was 15% or greater, evidence of gas leakage or irregular breathing.

LCI denoted the number of lung volume turnovers (cumulative expired volume divided by functional residual capacity) which entailed the reduction in end-tidal nitrogen concentration to 1/40^th^ of its initial concentration. Higher LCI denoted a greater magnitude of ventilation heterogeneity.

**Results**

**LCI and MMEF for discriminating moderate-to-severe bronchiectasis**

Overall, LCI and MMEF% predicted had similar power in discriminating moderate-to-severe bronchiectasis from mild bronchiectasis (Figure 2, Table S1). The diagnostic value of LCI and MMEF% predicted was higher for discriminating severe bronchiectasis alone [AUC: 0.705, 95%CI: (0.604, 0.806) for LCI; AUC: 0.670, 95%CI: (0.558, 0.782) for MMEF% predicted] compared with moderate-to-severe bronchiectasis [AUC: 0.672, 95%CI: (0.566, 0.778) for LCI; AUC: 0.629, 95%CI: (0.522, 0.736) for MMEF% predicted], as did in patients with HRCT score of 13 or greater [AUC: 0.920, 95%CI: (0.865, 0.975) for LCI; AUC: 0.813, 95%CI: (0.707, 0.919) for MMEF% predicted] compared with those who had HRCT score of 7 or greater [AUC: 0.834, 95%CI: (0.759, 0.910) for LCI; AUC: 0.821, 95%CI: (0.745, 0.897) for MMEF% predicted].

**FEV_1_ versus** **LCI and MMEF for discriminating moderate-to-severe bronchiectasis**

In previous studies, FEV_1_% predicted has been a major conventional spirometric parameter. It has also been suggested that LCI conferred greater diagnostic value than FEV_1_% predicted in the diagnosis of bronchiectasis. In this study, we have further compared the diagnostic performance of FEV_1_% predicted, LCI and MMEF% predicted.

As demonstrated in Figure E1-A, LCI (AUC: 0.672) did performed slightly superior to FEV_1_% predicted (AUC: 0.626) and MMEF% predicted (AUC: 0.629) for discriminating patients with moderate-to-severe bronchiectasis from those with mild bronchiectasis.

These findings applied for (AUC for LCI: 0.705, FEV_1_% predicted: 0.702, MMEF% predicted: 0.670) when discriminating patients with severe bronchiectasis from those with mild-to-moderate bronchiectasis (Figure E1-B).

**Diagnostic value of LCI and MMEF to discriminate patients with or without FEV_1_ >80% predicted**

Previous studies have reported that LCI more readily identify early-stage cystic fibrosis than FEV_1_, the most widely used spirometric parameter. Hence, in this study, we have also compared the diagnostic value of LCI and MMEF% predicted in discriminating patients with or without FEV_1_ >80% predicted. As shown in Table S2, MMEF% predicted conferred a higher assay specificity (0.97 vs. 0.80) and identical sensitivity (0.79 vs. 0.79) as compared with LCI. Hence, MMEF% predicted would at least be not inferior to LCI in identifying bronchiectasis with relatively preserved lung function.

**Correlation between LCI and MMEF in clinically stable bronchiectasis**

Following adjustment with age, sex and body-mass index, the LCI correlated negatively with MMEF% predicted (r= -0.64, P<.01), which was independent of disease severity as rated by the *BSI* (0-4 points: r= -0.61, P<.01; 5-8 points: r= -0.64, P<.01; 9 points or greater: r= -0.65, P<.01) and HRCT total score (0-6 points: r= -0.48, P<.01; 7-12 points: r= -0.71, P<.01) except for those with HRCT score of 13-18 (r= -0.12, P=.66). (Table 2)

However, when further adjusted with FEV_1_% predicted, the significant correlation existed only in patients with moderate bronchiectasis (r= -0.49, P<.01) and HRCT score of 7 or greater and 12 or lower (r= -0.43, P<.01) (Table S3).

**Correlation between LCI and FEV_1_/FVC% in different subgroups**

One may question the correlation existed only for LCI and MMEF% predicted, but not other spirometric parameters such as the FEV_1_/FVC%. To this end, we have also performed correlation analysis between LCI and FEV_1_/FVC% in different subgroups. According to Table S4, there was no significant correlation between LCI and FEV_1_/FVC%, regardless of the disease severity (all P>.05).

**Concordance of LCI and MMEF% predicted in reflecting different clinical characteristics of bronchiectasis patients with BSI <5**

Compared with MMEF% predicted, higher levels of LCI had consistently lower concordance to reflecting 3 or more bronchiectatic lobes and bilateral bronchiectasis.

However, higher levels of LCI had consistently higher concordance to reflecting HRCT total score of 9 or higher and cystic bronchiectasis. Both parameters had comparable ability in discriminating patients with *Pseudomonas aeruginosa* colonization and FEV_1_ of 80% predicted or lower.

Notably, none of the above difference reached statistical significance because the 95%CI for both variables was overlapped. (Table S5)

**Concordance of LCI and MMEF% predicted in reflecting different clinical characteristics of patients with BSI equal to or greater than 5 and lower than 9**

Compared with MMEF% predicted, higher levels of LCI had consistently higher concordance to reflecting 3 or more bronchiectatic lobes, HRCT total score of 9 or higher, bilateral bronchiectasis, and *Pseudomonas aeruginosa* colonization.

However, higher levels of LCI had consistently lower concordance to reflecting BSI of 5 or greater and FEV_1_ of 80% predicted or lower. Both parameters had comparable ability in discriminating patients with cystic bronchiectasis and ventilation heterogeneity.

None of the above difference reached statistical significance because the 95%CI for both variables was overlapped. (Table S6)

**Concordance of LCI and MMEF% predicted in reflecting different clinical characteristics of bronchiectasis patients with BSI ≥9**

Compared with MMEF% predicted, higher levels of LCI had higher concordance to reflecting cystic bronchiectasis.

However, higher levels of LCI had lower concordance to reflecting and FEV_1_ of 80% predicted or lower.

Both parameters had comparable ability in discriminating patients with 3 or more bronchiectatic lobes, HRCT total score of 9 or higher, BSI of 5 or greater, bilateral bronchiectasis, ventilation heterogeneity and *Pseudomonas aeruginosa* colonization.

None of the above difference reached statistical significance because the 95%CI for both variables was overlapped. (Table S7)

**Concordance of LCI and MMEF% predicted in reflecting different clinical characteristics of bronchiectasis patients with HRCT total score <7**

Compared with MMEF% predicted, higher levels of LCI had consistently higher concordance to reflecting 3 or more bronchiectatic lobes, HRCT total score of 9 or higher, BSI of 5 or greater, cystic bronchiectasis, and *Pseudomonas aeruginosa* colonization.

However, higher levels of LCI had consistently lower concordance to reflecting bilateral bronchiectasis and FEV_1_ of 80% predicted or lower. Both parameters had comparable ability in discriminating patients with ventilation heterogeneity.

None of the above difference reached statistical significance because the 95%CI for both variables was overlapped. (Table S8)

**Concordance of LCI and MMEF% predicted in reflecting different clinical characteristics of patients with HRCT total score equal to or greater than 7 and lower than 13**

Compared with MMEF% predicted, higher levels of LCI had consistently higher concordance to reflecting HRCT total score of 9 or higher, BSI of 5 or greater, *Pseudomonas aeruginosa* colonization, and ventilation heterogeneity.

However, higher levels of LCI had consistently lower concordance to reflecting cystic bronchiectasis and FEV_1_ of 80% predicted or lower.

Both parameters had comparable ability in discriminating patients with 3 or more bronchiectatic lobes and bilateral bronchiectasis.

None of the above difference reached statistical significance because the 95%CI for both variables was overlapped. (Table S9)

**Concordance of LCI and MMEF% predicted in reflecting different clinical characteristics of bronchiectasis patients with HRCT total score ≥13**

Higher levels of LCI had consistently lower concordance to reflecting BSI of 5 or greater, *Pseudomonas aeruginosa* colonization, and FEV_1_ of 80% predicted or lower.

The difference in discriminatory capacity could not be evaluated in this subgroup..

The findings in this subgroup should be interpreted with great caution and might not be generalizable for clinical application. (Table S10)

**Clinical variable attributes’ impacts on LCI and MMEF% predicted in patients with BSI <5**

Both LCI and MMEF% predicted were significantly correlated with the number of bronchiectatic lobes, HRCT total score, age, FEV_1_% predicted, and the presence of cystic bronchiectasis, ventilation heterogeneity, and bilateral bronchiectasis (all P<.001). The overall effect sizes were numerically greater for MMEF% predicted than for LCI. There was no correlation between LCI and sex or *Pseudomonas aeruginosa* colonization. However, MMEF% predicted also correlated significantly with sex and *Pseudomonas aeruginosa* colonization. (Table S11)

**Clinical variable attributes’ impacts on LCI and MMEF% predicted in patients with BSI equal to or greater than 5 and lower than 9**

Both LCI and MMEF% predicted were significantly correlated with HRCT total score, *Pseudomonas aeruginosa* colonization, and the presence of ventilation heterogeneity (all P<.001). The overall effect sizes were numerically greater for MMEF% predicted than for LCI.

MMEF% predicted, but not LCI, correlated significantly with the number of bronchiectatic lobes, age, FEV_1_% predicted, and the presence of cystic bronchiectasis and bilateral bronchiectasis (all P<.001). Neither MMEF% predicted nor LCI correlated with sex. (Table S12)

**Clinical variable attributes’ impacts on LCI and MMEF% predicted in patients with BSI ≥9**

Both LCI and MMEF% predicted were significantly correlated with HRCT total score, age, FEV_1_% predicted, sex, and the presence of cystic bronchiectasis, ventilation heterogeneity, and bilateral bronchiectasis (all P<.001). The overall effect sizes were numerically greater for MMEF% predicted than for LCI.

MMEF% predicted, but not LCI, correlated significantly with the number of bronchiectatic lobes and *Pseudomonas aeruginosa* colonization (all P<.001). (Table S13)

**Clinical variable attributes’ impacts on LCI and MMEF% predicted in patients with HRCT total score <7**

Both LCI and MMEF% predicted were significantly correlated with the number of bronchiectatic lobes, age, sex, FEV_1_% predicted, *Pseudomonas aeruginosa* colonization, and the presence of ventilation heterogeneity, and bilateral bronchiectasis (all P<.001). The overall effect sizes were numerically greater for MMEF% predicted than for LCI.

MMEF% predicted, but not LCI, correlated significantly with the HRCT total score and the presence of cystic bronchiectasis (both P<.001). (Table S14)

**Clinical variable attributes’ impacts on LCI and MMEF% predicted in patients with HRCT total score equal to or greater than 7 and lower than 13**

Both LCI and MMEF% predicted were significantly correlated with HRCT total score, FEV_1_% predicted, and the presence of cystic bronchiectasis (all P<.001). The overall effect sizes were numerically greater for MMEF% predicted than for LCI.

MMEF% predicted, but not LCI, correlated significantly with the number of bronchiectatic lobes, age, sex, *Pseudomonas aeruginosa* colonization, and the presence of ventilation heterogeneity, and bilateral bronchiectasis (all P<.001). (Table S15)

**Clinical variable attributes’ impacts on LCI and MMEF% predicted in patients with HRCT total score ≥13**

Both LCI and MMEF% predicted were significantly correlated with age, FEV_1_% predicted, sex, and the presence of cystic bronchiectasis (all P<.001). The overall effect sizes were numerically greater for MMEF% predicted than for LCI.

MMEF% predicted, but not LCI, correlated significantly with HRCT total score. However, LCI, but not MMEF% predicted, correlated significantly with *Pseudomonas aeruginosa* colonization. Neither parameter correlated significantly with the number of bronchiectatic lobes (both P>.05).

Nonetheless, these findings should be interpreted with great caution because of the limited sample sizes. (Table S16)

**Table S1 Diagnostic value of LCI and MMEF to discriminate patients with moderate to severe bronchiectasis**

|  | **Parameters** | **Area under curve** | **P value** | **95% confidence interval** | | **Cut-off** | **Sensitivity** | **Specificity** |
| --- | --- | --- | --- | --- | --- | --- | --- | --- |
|  |  |  |  | **Lower limit** | **Upper limit** |  |  |  |
| **BSI ≥5** | **LCI** | 0.672 | .003 | 0.566 | 0.778 | 16.38 | 0.500 | 0.816 |
|  | **MMEF% predicted** | 0.629 | .026 | 0.522 | 0.736 | 27.51 | 0.431 | 0.868 |
| **BSI ≥9** | **LCI** | 0.705 | .001 | 0.604 | 0.806 | 13.17 | 0.848 | 0.481 |
|  | **MMEF% predicted** | 0.670 | .005 | 0.558 | 0.782 | 26.05 | 0.576 | 0.792 |
| **HRCT score ≥7** | **LCI** | 0.834 | <.001 | 0.759 | 0.910 | 15.80 | 0.697 | 0.909 |
|  | **MMEF% predicted** | 0.821 | <.001 | 0.745 | 0.897 | 65.43 | 0.818 | 0.432 |
| **HRCT score ≥13** | **LCI** | 0.920 | <.001 | 0.865 | 0.975 | 17.02 | 0.950 | 0.833 |
|  | **MMEF% predicted** | 0.813 | <.001 | 0.707 | 0.919 | 27.51 | 0.800 | 0.778 |

**Table S2 Diagnostic value of LCI and MMEF to discriminate patients with or without FEV_1_** >80% predicted

| **Parameters** | **Area under curve** | **P value** | **95% confidence interval** | | **Cut-off** | **Sensitivity** | **Specificity** |
| --- | --- | --- | --- | --- | --- | --- | --- |
|  |  |  | **Lower limit** | **Upper limit** |  |  |  |
| **LCI** | 0.80 | <0.01 | 0.71 | 0.89 | 13.1 | 0.79 | 0.80 |
| **MMEF% predicted** | 0.93 | <0.01 | 0.88 | 0.98 | 50.3 | 0.79 | 0.97 |

**Table S3 Comparison and correlation of LCI and MMEF% predicted in different subgroups, adjusting for the age, sex, body-mass index and the baseline FEV_1_**% predicted

|  | **No.** | **LCI**  **Median (95%CI)** | **MMEF% predicted**  **Median (95%CI)** | **r Value of Correlation #** | **P Value of Correlation #** |
| --- | --- | --- | --- | --- | --- |
| **All patients** | 110 | 14.70 (14.49, 16.13) | 45.30 (41.72, 52.72) | -0.21 | .04 |
| **0≤ BSI <5** | 38 | 12.90 (12.57, 15.05) | 50.64 (46.06, 65.23) | -0.12 | .48 |
| **5≤ BSI ≤8** | 39 | 14.60 (13.68, 15.96) | 47.02 (39.43, 58.72) | -0.49 | <.01 |
| **BSI ≥9** | 33 | 16.90 (15.89, 19.37) | 22.87 (26.55, 44.09) | -0.14 | .48 |
| **P value *** | - | <.01 | <.01 | - | - |
| **1≤ HRCT score <7** | 44 | 12.40 (11.84, 13.18) | 66.14 (58.26, 74.01) | -0.29 | .07 |
| **7≤ HRCT score ≤12** | 46 | 16.20 (14.60, 16.69) | 34.26 (32.11, 46.61) | -0.43 | <.01 |
| **HRCT score ≥13** | 20 | 19.15 (18.73, 22.69) | 16.32 (14.78, 32.57) | -0.22 | .42 |
| **P value **** | - | <.01 | <.01 | - | - |

95%CI: 95% confidence interval

LCI: lung clearance index; MMEF: maximal mid-expiratory flow

*Comparison among patients with BSI less than 5, BSI of 5 or greater and 8 or lower, and those with BSI of 9 or greater.

** Comparison among patients with HRCT total score less than 7, HRCT total score of 7 or greater and 12 or lower, and those with HRCT total score of 13 or greater.

# Correlation between LCI and MMEF% predicted in different subgroups was analyzed with partial correlation model, adjusting for the age, sex, body-mass index and the baseline FEV_1_% predicted.

**Table S4 Correlation between LCI and FEV_1_**/FVC% in different subgroups

|  | **No.** | **r Value of Correlation #** | **P Value of Correlation #** |
| --- | --- | --- | --- |
| **All patients** | 110 | -0.15 | .13 |
| **0≤ BSI <5** | 38 | -0.14 | .94 |
| **5≤ BSI ≤8** | 39 | -0.33 | .05 |
| **BSI ≥9** | 33 | -0.10 | .62 |
| **P value *** | - | - | - |
| **1≤ HRCT score <7** | 44 | -0.27 | .09 |
| **7≤ HRCT score ≤12** | 46 | -0.26 | .10 |
| **HRCT score ≥13** | 20 | -0.27 | .32 |
| **P value **** | - | - | - |

95%CI: 95% confidence interval

LCI: lung clearance index

*Comparison among patients with BSI less than 5, BSI of 5 or greater and 8 or lower, and those with BSI of 9 or greater.

** Comparison among patients with HRCT total score less than 7, HRCT total score of 7 or greater and 12 or lower, and those with HRCT total score of 13 or greater.

# Correlation between LCI and MMEF% predicted in different subgroups was analyzed with partial correlation model, adjusting for the age, sex, body-mass index and the baseline FEV_1_% predicted.

**Table S5 Concordance of lung clearance index and maximal mid-expiratory flow with clinical variables in bronchiectasis patients with BSI <**5

|  | **LCI** | | | |  | **MMEF% predicted** | | | |
| --- | --- | --- | --- | --- | --- | --- | --- | --- | --- |
|  | **Lower 50^th^ percentile*** | **Upper 50^th^ percentile*** | **Total** | **Concordance (95%CI)** |  | **Lower 50^th^ percentile**** | **Upper 50^th^ percentile**** | **Total** | **Concordance (95%CI)** |
| **≤3 bronchiectatic lobes** | 13 | 4 | 17 | 0.368  (0.047, 0.689) |  | 19 | 4 | 23 | 0.559  (0.289, 0.829) |
| **>3 bronchiectatic lobes** | 6 | 9 | 15 |  |  | 4 | 11 | 15 |  |
| **Total** | 19 | 13 | 32 | - |  | 23 | 15 | 38 | - |
| **HRCT total score ≤9** | 24 | 8 | 32 | 0.396  (0.100, 0.692) |  | 21 | 11 | 32 | 0.201  (-0.077, 0.479) |
| **HRCT total score >9** | 1 | 5 | 6 |  |  | 2 | 4 | 6 |  |
| **Total** | 25 | 13 | 38 | - |  | 23 | 15 | 38 | - |
| **BSI ≤5** | 25 | 13 | 38 | NA |  | 15 | 23 | 38 | NA |
| **BSI >5** | 0 | 0 | 0 |  |  | 0 | 0 | 0 |  |
| **Total** | 25 | 13 | 38 | - |  | 15 | 23 | 38 | - |
| **Unilateral bronchiectasis** | 12 | 1 | 13 | 0.330  (0.099, 0.561) |  | 13 | 0 | 13 | 0.506  (0.277, 0.735) |
| **Bilateral bronchiectasis** | 13 | 12 | 25 |  |  | 10 | 15 | 25 |  |
| **Total** | 25 | 13 | 38 | - |  | 23 | 15 | 38 | - |
| **Tubular/varicose bronchiectasis** | 11 | 4 | 15 | 0.112  (-0.158, 0.382) |  | 9 | 6 | 15 | -0.008  (-0.300, 0.284) |
| **Cystic bronchiectasis** | 14 | 9 | 23 |  |  | 14 | 9 | 23 |  |
| **Total** | 25 | 13 | 38 | - |  | 23 | 15 | 38 | - |
| **Homogeneity** | 13 | 1 | 14 | 0.368  (0.131, 0.605) |  | 10 | 4 | 14 | 0.152  (-0.124, 0.428) |
| **Heterogeneity** | 12 | 12 | 24 |  |  | 13 | 11 | 24 |  |
| **Total** | 25 | 13 | 38 | - |  | 23 | 15 | 38 | - |
| **No *P. aeruginosa* colonized** | 22 | 12 | 34 | -0.052  (-0.283, 0.179) |  | 20 | 14 | 34 | -0.073  (-0.287, 0.141) |
| ***P. aeruginosa* colonized** | 3 | 1 | 4 |  |  | 3 | 1 | 4 |  |
| **Total** | 25 | 13 | 38 | - |  | 25 | 13 | 38 | - |
| **FEV_1_ predicted ≤80%** | 12 | 0 | 12 | 0.387  (0.173, 0.601) |  | 11 | 1 | 12 | 0.365  (0.126, 0.604) |
| **FEV_1_ predicted >80%** | 13 | 13 | 26 |  |  | 12 | 14 | 26 |  |
| **Total** | 25 | 13 | 38 | - |  | 23 | 15 | 38 | - |

Data are presented with counts unless otherwise stated.

95%CI: 95% confidence interval

LCI: lung clearance index; MMEF: maximal mid-expiratory flow

* For LCI, “Low” denoted the values being equal to or lower than the median (14.70), whereas “high” indicated the values being higher than the median (14.70).

** For MMEF% predicted, “Low” denoted the values being equal to or lower than the median (45.3%), whereas “high” indicated the values being higher than the median (45.3%).

**Table S6 Concordance of lung clearance index and maximal mid-expiratory flow with clinical variables in bronchiectasis patients with BSI equal to or greater than 5 and lower than** 9

|  | **LCI** | | | |  | **MMEF% predicted** | | | |
| --- | --- | --- | --- | --- | --- | --- | --- | --- | --- |
|  | **Lower 50^th^ percentile*** | **Upper 50^th^ percentile*** | **Total** | **Concordance (95%CI)** |  | **Lower 50^th^ percentile**** | **Upper 50^th^ percentile**** | **Total** | **Concordance (95%CI)** |
| **≤3 bronchiectatic lobes** | 12 | 1 | 13 | 0.542  (0.297, 0.787) |  | 10 | 3 | 13 | 0.262  (-0.005, 0.529) |
| **>3 bronchiectatic lobes** | 8 | 18 | 26 |  |  | 12 | 14 | 26 |  |
| **Total** | 20 | 19 | 39 | - |  | 22 | 17 | 39 | - |
| **HRCT total score ≤9** | 19 | 9 | 28 | 0.481  (0.228, 0.734) |  | 19 | 9 | 28 | 0.348  (0.062, 0.634) |
| **HRCT total score >9** | 1 | 10 | 11 |  |  | 3 | 8 | 11 |  |
| **Total** | 20 | 19 | 39 | - |  | 22 | 17 | 39 | - |
| **BSI ≤5** | 8 | 3 | 11 | 0.239  (-0.031, 0.509) |  | 9 | 2 | 11 | 0.271  (0.022, 0.520) |
| **BSI >5** | 12 | 16 | 28 |  |  | 13 | 15 | 28 |  |
| **Total** | 20 | 19 | 39 | - |  | 22 | 17 | 39 | - |
| **Unilateral bronchiectasis** | 5 | 1 | 6 | 0.194  (-0.022, 0.410) |  | 4 | 2 | 6 | 0.058  (-0.144, 0.260) |
| **Bilateral bronchiectasis** | 15 | 18 | 33 |  |  | 18 | 15 | 33 |  |
| **Total** | 20 | 19 | 39 | - |  | 22 | 17 | 39 | - |
| **Tubular/varicose bronchiectasis** | 11 | 3 | 14 | 0.389  (0.115, 0.663) |  | 12 | 2 | 14 | 0.406  (0.147, 0.665) |
| **Cystic bronchiectasis** | 9 | 16 | 25 |  |  | 10 | 15 | 25 |  |
| **Total** | 20 | 19 | 39 | - |  | 22 | 17 | 39 | - |
| **Homogeneity** | 9 | 2 | 11 | 0.341  (0.080, 0.602) |  | 10 | 1 | 11 | 0.369  (0.130, 0.608) |
| **Heterogeneity** | 11 | 17 | 28 |  |  | 12 | 16 | 28 |  |
| **Total** | 20 | 19 | 39 | - |  | 22 | 17 | 39 | - |
| **No *P. aeruginosa* colonized** | 19 | 12 | 31 | 0.323  (0.078, 0.568) |  | 20 | 11 | 31 | 0.279  (0.007, 0.551) |
| ***P. aeruginosa* colonized** | 1 | 7 | 8 |  |  | 2 | 6 | 8 |  |
| **Total** | 20 | 19 | 39 | - |  | 22 | 17 | 39 | - |
| **FEV_1_ predicted ≤80%** | 9 | 3 | 12 | 0.289  (0.017, 0.561) |  | 12 | 0 | 12 | 0.511  (0.284, 0.739) |
| **FEV_1_ predicted >80%** | 11 | 16 | 27 |  |  | 10 | 17 | 27 |  |
| **Total** | 20 | 19 | 39 | - |  | 22 | 17 | 39 | - |

Data are presented with counts unless otherwise stated.

95%CI: 95% confidence interval

LCI: lung clearance index; MMEF: maximal mid-expiratory flow

NA: Not applicable

* For LCI, “Low” denoted the values being equal to or lower than the median (14.70), whereas “high” indicated the values being higher than the median (14.70).

** For MMEF% predicted, “Low” denoted the values being equal to or lower than the median (45.3%), whereas “high” indicated the values being higher than the median (45.3%).

**Table S7 Concordance of lung clearance index and maximal mid-expiratory flow with clinical variables in bronchiectasis patients with BSI** ≥9

|  | **LCI** | | | |  | **MMEF% predicted** | | | |
| --- | --- | --- | --- | --- | --- | --- | --- | --- | --- |
|  | **Lower 50^th^ percentile*** | **Upper 50^th^ percentile*** | **Total** | **Concordance (95%CI)** |  | **Lower 50^th^ percentile**** | **Upper 50^th^ percentile**** | **Total** | **Concordance (95%CI)** |
| **≤3 bronchiectatic lobes** | 4 | 1 | 5 | 0.415  (0.078, 0.752) |  | 4 | 1 | 5 | 0.415  (0.078, 0.752) |
| **>3 bronchiectatic lobes** | 6 | 22 | 28 |  |  | 6 | 22 | 28 |  |
| **Total** | 10 | 23 | 33 | - |  | 10 | 23 | 33 | - |
| **HRCT total score ≤9** | 8 | 5 | 13 | 0.537  (0.241, 0.833) |  | 8 | 5 | 13 | 0.537  (0.241, 0.833) |
| **HRCT total score >9** | 2 | 18 | 20 |  |  | 2 | 18 | 20 |  |
| **Total** | 10 | 23 | 33 | - |  | 10 | 23 | 33 | - |
| **BSI ≤5** | 0 | 0 | 0 | NA |  | 0 | 0 | 0 | NA |
| **BSI >5** | 10 | 23 | 33 |  |  | 10 | 23 | 33 |  |
| **Total** | 10 | 23 | 33 | - |  | 10 | 23 | 33 | - |
| **Unilateral bronchiectasis** | 1 | 0 | 1 | 0.134  (-0.107, 0.375) |  | 1 | 0 | 1 | 0.134  (-0.107, 0.375) |
| **Bilateral bronchiectasis** | 9 | 23 | 32 |  |  | 9 | 23 | 32 |  |
| **Total** | 10 | 23 | 33 | - |  | 10 | 23 | 33 | - |
| **Tubular/varicose bronchiectasis** | 3 | 2 | 5 | 0.248  (-0.097, 0.593) |  | 2 | 3 | 5 | 0.081  (-0.248, 0.410) |
| **Cystic bronchiectasis** | 7 | 21 | 28 |  |  | 8 | 20 | 28 |  |
| **Total** | 10 | 23 | 33 | - |  | 10 | 23 | 33 | - |
| **Homogeneity** | 3 | 3 | 6 | 0.191  (-0.158, 0.540) |  | 3 | 3 | 6 | 0.191  (-0.158, 0.540) |
| **Heterogeneity** | 7 | 20 | 27 |  |  | 7 | 20 | 27 |  |
| **Total** | 10 | 23 | 33 | - |  | 10 | 23 | 33 | - |
| **No *P. aeruginosa* colonized** | 4 | 10 | 14 | -0.031  (-0.358, 0.297) |  | 4 | 10 | 14 | -0.031  (-0.358, 0.297) |
| ***P. aeruginosa* colonized** | 6 | 13 | 19 |  |  | 6 | 13 | 19 |  |
| **Total** | 10 | 23 | 33 | - |  | 10 | 23 | 33 | - |
| **FEV_1_ predicted ≤80%** | 4 | 2 | 6 | 0.353  (0.006, 0.700) |  | 6 | 0 | 6 | 0.676  (0.396, 0.956) |
| **FEV_1_ predicted >80%** | 6 | 21 | 27 |  |  | 4 | 23 | 27 |  |
| **Total** | 10 | 23 | 33 | - |  | 10 | 23 | 33 | - |

Data are presented with counts unless otherwise stated.

95%CI: 95% confidence interval

LCI: lung clearance index; MMEF: maximal mid-expiratory flow

NA: Not applicable

* For LCI, “Low” denoted the values being equal to or lower than the median (14.70), whereas “high” indicated the values being higher than the median (14.70).

** For MMEF% predicted, “Low” denoted the values being equal to or lower than the median (45.3%), whereas “high” indicated the values being higher than the median (45.3%).

**Table S8 Concordance of lung clearance index and maximal mid-expiratory flow with clinical variables in bronchiectasis patients with HRCT total score <**7

|  | **LCI** | | | |  | **MMEF% predicted** | | | |
| --- | --- | --- | --- | --- | --- | --- | --- | --- | --- |
|  | **Lower 50^th^ percentile*** | **Upper 50^th^ percentile*** | **Total** | **Concordance (95%CI)** |  | **Lower 50^th^ percentile**** | **Upper 50^th^ percentile**** | **Total** | **Concordance (95%CI)** |
| **≤3 bronchiectatic lobes** | 33 | 4 | 37 | 0.438  (0.089, 0.787) |  | 31 | 6 | 37 | 0.349  (0.014, 0.684) |
| **>3 bronchiectatic lobes** | 3 | 4 | 7 |  |  | 3 | 4 | 7 |  |
| **Total** | 36 | 8 | 44 | - |  | 34 | 10 | 44 | - |
| **HRCT total score ≤9** | 36 | 8 | 44 | NA |  | 34 | 10 | 44 | NA |
| **HRCT total score >9** | 0 | 0 | 0 |  |  | 0 | 0 | 0 |  |
| **Total** | 36 | 8 | 44 | - |  | 34 | 10 | 44 | - |
| **BSI ≤5** | 25 | 4 | 29 | 0.145  (-0.141, 0.431) |  | 23 | 6 | 29 | 0.065  (-0.225, 0.355) |
| **BSI >5** | 11 | 4 | 15 |  |  | 11 | 4 | 15 |  |
| **Total** | 36 | 8 | 44 | - |  | 34 | 10 | 44 | - |
| **Unilateral bronchiectasis** | 17 | 2 | 19 | 0.122  (-0.078, 0.322) |  | 17 | 2 | 19 | 0.196  (-0.020, 0.412) |
| **Bilateral bronchiectasis** | 19 | 6 | 25 |  |  | 17 | 8 | 25 |  |
| **Total** | 36 | 8 | 44 | - |  | 34 | 10 | 44 | - |
| **Tubular/varicose bronchiectasis** | 19 | 3 | 22 | 0.091  (-0.136, 0.318) |  | 16 | 6 | 22 | -0.091 (-0.338, 0.156) |
| **Cystic bronchiectasis** | 17 | 5 | 22 |  |  | 18 | 4 | 22 |  |
| **Total** | 36 | 8 | 44 | - |  | 34 | 10 | 44 | - |
| **Homogeneity** | 21 | 1 | 22 | 0.273  (0.053, 0.493) |  | 20 | 2 | 22 | 0.273  (0.034, 0.512) |
| **Heterogeneity** | 15 | 7 | 22 |  |  | 14 | 8 | 22 |  |
| **Total** | 36 | 8 | 44 | - |  | 34 | 10 | 44 | - |
| **No *P. aeruginosa* colonized** | 30 | 6 | 36 | 0.083  (-0.240, 0.406) |  | 28 | 6 | 34 | 0.025  (-0.294, 0.344) |
| ***P. aeruginosa* colonized** | 6 | 2 | 8 |  |  | 8 | 2 | 10 |  |
| **Total** | 36 | 8 | 44 | - |  | 36 | 8 | 44 | - |
| **FEV_1_ predicted ≤80%** | 19 | 2 | 21 | 0.161  (-0.055, 0.377) |  | 20 | 1 | 21 | 0.335  (0.112, 0.558) |
| **FEV_1_ predicted >80%** | 17 | 6 | 23 |  |  | 14 | 9 | 23 |  |
| **Total** | 36 | 8 | 44 | - |  | 34 | 10 | 44 | - |

Data are presented with counts unless otherwise stated.

95%CI: 95% confidence interval

LCI: lung clearance index; MMEF: maximal mid-expiratory flow

* For LCI, “Low” denoted the values being equal to or lower than the median (14.70), whereas “high” indicated the values being higher than the median (14.70).

** For MMEF% predicted, “Low” denoted the values being equal to or lower than the median (45.3%), whereas “high” indicated the values being higher than the median (45.3%).

**Table S9 Concordance of lung clearance index and maximal mid-expiratory flow with clinical variables in bronchiectasis patients with HRCT total score equal to or greater than 7 and lower than 13**

|  | **LCI** | | | |  | **MMEF% predicted** | | | |
| --- | --- | --- | --- | --- | --- | --- | --- | --- | --- |
|  | **Lower 50^th^ percentile*** | **Upper 50^th^ percentile*** | **Total** | **Concordance (95%CI)** |  | **Lower 50^th^ percentile**** | **Upper 50^th^ percentile**** | **Total** | **Concordance (95%CI)** |
| **≤3 bronchiectatic lobes** | 2 | 2 | 4 | 0.046  (-0.156, 0.248) |  | 2 | 2 | 4 | 0.058  (-1.063, 1.179) |
| **>3 bronchiectatic lobes** | 16 | 26 | 42 |  |  | 15 | 27 | 42 |  |
| **Total** | 18 | 28 | 46 | - |  | 17 | 29 | 46 | - |
| **HRCT total score ≤9** | 15 | 14 | 29 | 0.301  (0.062, 0.540) |  | 14 | 15 | 29 | 0.267  (0.032, 0.502) |
| **HRCT total score >9** | 3 | 14 | 17 |  |  | 3 | 14 | 17 |  |
| **Total** | 18 | 28 | 46 | - |  | 17 | 29 | 46 | - |
| **BSI ≤5** | 8 | 9 | 17 | 0.124  (-0.166, 0.414) |  | 7 | 10 | 17 | 0.067  (-0.223, 0.357) |
| **BSI >5** | 10 | 19 | 29 |  |  | 10 | 19 | 29 |  |
| **Total** | 18 | 28 | 46 | - |  | 17 | 29 | 46 | - |
| **Unilateral bronchiectasis** | 1 | 0 | 1 | 0.067  (-0.060, 0.194) |  | 1 | 0 | 1 | 0.073  (-0.064, 0.210) |
| **Bilateral bronchiectasis** | 17 | 28 | 45 |  |  | 16 | 29 | 45 |  |
| **Total** | 18 | 28 | 46 | - |  | 17 | 29 | 46 | - |
| **Tubular/varicose bronchiectasis** | 6 | 5 | 11 | 0.166  (-0.113, 0.444) |  | 7 | 4 | 11 | 0.295  (0.015, 0.575) |
| **Cystic bronchiectasis** | 12 | 23 | 35 |  |  | 10 | 25 | 35 |  |
| **Total** | 18 | 28 | 46 | - |  | 17 | 29 | 46 | - |
| **Homogeneity** | 4 | 5 | 9 | 0.048  (-0.215, 0.311) |  | 3 | 6 | 9 | -0.034  (-0.293, 0.225) |
| **Heterogeneity** | 14 | 23 | 37 |  |  | 14 | 23 | 37 |  |
| **Total** | 18 | 28 | 46 | - |  | 17 | 29 | 46 | - |
| **No *P. aeruginosa* colonized** | 14 | 17 | 31 | 0.151  (-0.086, 0.388) |  | 13 | 18 | 31 | 0.123  (-0.109, 0.354) |
| ***P. aeruginosa* colonized** | 4 | 11 | 15 |  |  | 4 | 11 | 15 |  |
| **Total** | 18 | 28 | 46 | - |  | 17 | 29 | 46 | - |
| **FEV_1_ predicted ≤80%** | 6 | 2 | 8 | 0.291  (0.032, 0.550) |  | 8 | 0 | 8 | 0.528  (0.285, 0.771) |
| **FEV_1_ predicted >80%** | 12 | 26 | 38 |  |  | 9 | 29 | 38 |  |
| **Total** | 18 | 28 | 46 | - |  | 17 | 29 | 46 | - |

Data are presented with counts unless otherwise stated.

95%CI: 95% confidence interval

LCI: lung clearance index; MMEF: maximal mid-expiratory flow

* For LCI, “Low” denoted the values being equal to or lower than the median (14.70), whereas “high” indicated the values being higher than the median (14.70).

** For MMEF% predicted, “Low” denoted the values being equal to or lower than the median (45.3%), whereas “high” indicated the values being higher than the median (45.3%).

**Table S10 Concordance of lung clearance index and maximal mid-expiratory fl**ow with clinical variables in bronchiectasis patients with HRCT total score ≥13

|  | **LCI** | | | |  | **MMEF% predicted** | | | |
| --- | --- | --- | --- | --- | --- | --- | --- | --- | --- |
|  | **Lower 50^th^ percentile*** | **Upper 50^th^ percentile*** | **Total** | **Concordance (95%CI)** |  | **Lower 50^th^ percentile**** | **Upper 50^th^ percentile**** | **Total** | **Concordance (95%CI)** |
| **≤3 bronchiectatic lobes** | 0 | 0 | 0 | NA |  | 0 | 0 | 0 | NA |
| **>3 bronchiectatic lobes** | 1 | 19 | 20 |  |  | 4 | 16 | 20 |  |
| **Total** | 1 | 19 | 20 | - |  | 4 | 16 | 20 | - |
| **HRCT total score ≤9** | 0 | 0 | 0 | NA |  | 0 | 0 | 0 | NA |
| **HRCT total score >9** | 1 | 19 | 20 |  |  | 4 | 16 | 20 |  |
| **Total** | 1 | 19 | 20 | - |  | 4 | 16 | 20 | - |
| **BSI ≤5** | 0 | 3 | 3 | -0.081  (-0.206, 0.044) |  | 2 | 1 | 3 | 0.483  (-0.015, 0.981) |
| **BSI >5** | 1 | 16 | 17 |  |  | 2 | 15 | 17 |  |
| **Total** | 1 | 19 | 20 | - |  | 4 | 16 | 20 | - |
| **Unilateral bronchiectasis** | 0 | 0 | 0 | NA |  | 0 | 0 | 0 | NA |
| **Bilateral bronchiectasis** | 1 | 19 | 20 |  |  | 4 | 16 | 20 |  |
| **Total** | 1 | 19 | 20 | - |  | 4 | 16 | 20 | - |
| **Tubular/varicose bronchiectasis** | 0 | 1 | 1 | -0.053  (-0.126, 0.020) |  | 0 | 1 | 1 | -0.087  (0.230, 0.056) |
| **Cystic bronchiectasis** | 1 | 18 | 19 |  |  | 4 | 15 | 19 |  |
| **Total** | 1 | 19 | 20 | - |  | 4 | 16 | 20 | - |
| **Homogeneity** | 0 | 0 | 0 | NA |  | 0 | 0 | 0 | NA |
| **Heterogeneity** | 1 | 19 | 20 |  |  | 4 | 16 | 20 |  |
| **Total** | 1 | 19 | 20 | - |  | 4 | 16 | 20 | - |
| **No *P. aeruginosa* colonized** | 1 | 11 | 12 | 0.068  (-0.065, 0.201) |  | 3 | 9 | 12 | 0.107  (-0.185, 0.399) |
| ***P. aeruginosa* colonized** | 0 | 8 | 8 |  |  | 1 | 7 | 8 |  |
| **Total** | 1 | 19 | 20 | - |  | 4 | 16 | 20 | - |
| **FEV_1_ predicted ≤80%** | 0 | 1 | 1 | -0.053  (-0.126, 0.020) |  | 1 | 0 | 1 | 0.348  (0.167, 0.863) |
| **FEV_1_ predicted >80%** | 1 | 18 | 19 |  |  | 3 | 16 | 19 |  |
| **Total** | 1 | 19 | 20 | - |  | 4 | 16 | 20 | - |

Data are presented with counts unless otherwise stated.

95%CI: 95% confidence interval

LCI: lung clearance index; MMEF: maximal mid-expiratory flow

NA: not applicable

* For LCI, “Low” denoted the values being equal to or lower than the median (14.70), whereas “high” indicated the values being higher than the median (14.70).

** For MMEF% predicted, “Low” denoted the values being equal to or lower than the median (45.3%), whereas “high” indicated the values being higher than the median (45.3%).

**Table S11 Fixed-effect estimates in multivariate linear mixed model of the clinical variable attributes’ impacts on lung clearance index and maximal mid-expiratory flow in patients with BSI <**5

|  | **LCI** | | |  | **MMEF% predicted** | | |
| --- | --- | --- | --- | --- | --- | --- | --- |
|  | **Estimate** | **P value** | **95% CI** |  | **Estimate** | **P value** | **95% CI** |
| **Intercept** | **21.936** | **<.001** | **18.577, 25.295** |  | **-46.878** | **<.001** | **-50.237, -43.519** |
| **No. of bronchiectatic lobes** | **-1.369** | **<.001** | **-1.854, -0.883** |  | **-4.441** | **<.001** | **-4.926, -3.955** |
| **HRCT total score*** | **0.740** | **<.001** | **0.526, 0.954** |  | **2.359** | **<.001** | **2.145, 2.573** |
| **Age** | 0.010 | .523 | -0.021, 0.042 |  | **0.293** | **<.001** | **0.261, 0.325** |
| **FEV_1_ predicted%** | **-0.137** | **<.001** | **-0.161, -0.113** |  | **1.129** | **<.001** | **1.105, 1.153** |
| **Sex** | - | - | - |  | - | - | - |
| Males | Reference | Reference | Reference |  | Reference | Reference | Reference |
| Females | **0.906** | **.017** | **0.165, 1.646** |  | **2.837** | **<.001** | **2.096, 3.578** |
| **Sputum bacteriology** | - | - | - |  | - | - | - |
| *P. aeruginosa* colonized | Reference | Reference | Reference |  | Reference | Reference | Reference |
| No *P. aeruginosa* colonized | 0.478 | .413 | -0.666, 1.622 |  | **7.160** | **<.001** | **6.016, 8.304** |
| **Cystic bronchiectasis** | - | - | - |  | - | - | - |
| Yes | Reference | Reference | Reference |  | Reference | Reference | Reference |
| No | **2.305** | **<.001** | **1.448, 3.162** |  | **-4.521** | **<.001** | **-5.378, -3.664** |
| **Heterogeneity** | - | - | - |  | - | - | - |
| Yes | Reference | Reference | Reference |  | Reference | Reference | Reference |
| No | **-1.209** | **.003** | **-2.011, -0.407** |  | **2.445** | **<.001** | **1.643, 3.246** |
| **Bilateral bronchiectasis** | - | - | - |  | - | - | - |
| Yes | Reference | Reference | Reference |  | Reference | Reference | Reference |
| No | **-1.087** | **.025** | **-2.035, -0.139** |  | **2.381** | **<.001** | **1.433, 3.329** |

95%CI: 95% confidence interval, LCI: lung clearance index; MMEF: maximal mid-expiratory flow

Data in bold indicated the statistical analyses with significance.

*Modified Reiff score

**Table S12 Fixed-effect estimates in multivariate linear mixed model of the clinical variable attributes’ impacts on lung clearance index and maximal mid-expiratory flow in patients with BSI equal to or greater than 5 and lower than 9**

|  | **LCI** | | |  | **MMEF% predicted** | | |
| --- | --- | --- | --- | --- | --- | --- | --- |
|  | **Estimate** | **P value** | **95% CI** |  | **Estimate** | **P value** | **95% CI** |
| **Intercept** | **14.435** | **<.001** | **12.037, 16.832** |  | **-16.660** | **<.001** | **-19.058, -14.263** |
| **No. of bronchiectatic lobes** | -0.186 | .442 | -0.661, 0.289 |  | **0.989** | **<.001** | **0.514, 1.464** |
| **HRCT total score*** | **0.447** | **<.001** | **0.221, 0.672** |  | **-0.944** | **<.001** | **-1.170, -0.718** |
| **Age** | 0.021 | .087 | -0.003, 0.046 |  | **-0.044** | **<.001** | **-0.068, -0.019** |
| **FEV_1_ predicted%** | -0.017 | .130 | -0.039, 0.005 |  | **1.047** | **<.001** | **1.025, 1.069** |
| **Sex** | - | - | - |  | - | - | - |
| Males | Reference | Reference | Reference |  | Reference | Reference | Reference |
| Females | -0.048 | .903 | -0.814, 0.719 |  | -0.705 | .071 | -1.471, 0.061 |
| **Sputum bacteriology** | - | - | - |  | - | - | - |
| *P. aeruginosa* colonized | Reference | Reference | Reference |  | Reference | Reference | Reference |
| No *P. aeruginosa* colonized | **-2.333** | **<.001** | **-3.250, -1.415** |  | **1.009** | **.031** | **0.092, 1.926** |
| **Cystic bronchiectasis** | - | - | - |  | - | - | - |
| Yes | Reference | Reference | Reference |  | Reference | Reference | Reference |
| No | 0.420 | .384 | -0.525, 1.365 |  | **-9.929** | **<.001** | **-10.874, -8.984** |
| **Heterogeneity** | - | - | - |  | - | - | - |
| Yes | Reference | Reference | Reference |  | Reference | Reference | Reference |
| No | **-1.436** | **.002** | **-2.326, -0.546** |  | **10.533** | **<.001** | **9.643, 11.423** |
| **Bilateral bronchiectasis** | - | - | - |  | - | - | - |
| Yes | Reference | Reference | Reference |  | Reference | Reference | Reference |
| No | -0.298 | .622 | -1.486, 0.889 |  | **-2.305** | **<.001** | **-3.492, -1.117** |

95%CI: 95% confidence interval, LCI: lung clearance index; MMEF: maximal mid-expiratory flow

Data in bold indicated the statistical analyses with significance.

*Modified Reiff score

**Table S13 Fixed-effect estimates in multivariate linear mixed mode**l of the clinical variable attributes’ impacts on lung clearance index and maximal mid-expiratory flow in patients with BSI ≥9

|  | **LCI** | | |  | **MMEF% predicted** | | |
| --- | --- | --- | --- | --- | --- | --- | --- |
|  | **Estimate** | **P value** | **95% CI** |  | **Estimate** | **P value** | **95% CI** |
| **Intercept** | **8.936** | **<.001** | **5.499, 12.372** |  | **9.706** | **<.001** | **6.269, 13.143** |
| **No. of bronchiectatic lobes** | 0.345 | .204 | -0.187, 0.877 |  | **-4.345** | **<.001** | **-4.878, -3.814** |
| **HRCT total score*** | **0.598** | **<.001** | **0.430, 0.766** |  | **0.525** | **<.001** | **0.357, 0.693** |
| **Age** | **0.155** | **<.001** | **0.127, 0.184** |  | **-0.120** | **<.001** | **-0.149, -0.092** |
| **FEV_1_ predicted%** | **-0.139** | **<.001** | **-0.163, -0.116** |  | **1.051** | **<.001** | **1.027, 1.074** |
| **Sex** | - | - | - |  | - | - | - |
| Males | Reference | Reference | Reference |  | Reference | Reference | Reference |
| Females | **0.802** | **.044** | **0.023, 1.582** |  | **-2.265** | **<.001** | **-3.044, -1.486** |
| **Sputum bacteriology** | - | - | - |  | - | - | - |
| *P. aeruginosa* colonized | Reference | Reference | Reference |  | Reference | Reference | Reference |
| No *P. aeruginosa* colonized | -0.012 | .975 | -0.731, 0.708 |  | **-7.278** | **<.001** | **-7.998, -6.559** |
| **Cystic bronchiectasis** | - | - | - |  | - | - | - |
| Yes | Reference | Reference | Reference |  | Reference | Reference | Reference |
| No | **-1.618** | **.003** | **-2.698, -0.539** |  | **-2.338** | **<.001** | **-3.419, -1.259** |
| **Heterogeneity** | - | - | - |  | - | - | - |
| Yes | Reference | Reference | Reference |  | Reference | Reference | Reference |
| No | **2.967** | **<.001** | **1.724, 4.210** |  | **-17.995** | **<.001** | **-19.239, -16.752** |
| **Bilateral bronchiectasis** | - | - | - |  | - | - | - |
| Yes | Reference | Reference | Reference |  | Reference | Reference | Reference |
| No | **5.378** | **<.001** | **2.787, 7.970** |  | **-12.118** | **<.001** | **-14.709, -9.526** |

95%CI: 95% confidence interval, LCI: lung clearance index; MMEF: maximal mid-expiratory flow

Data in bold indicated the statistical analyses with significance.

*Modified Reiff score

**Table S14 Fixed-effect estimates in multivariate linear mixed model of the clinical variable attributes’ impacts on lung clearance index and maximal mid-expiratory flow in patients with HRCT total score <7**

|  | **LCI** | | |  | **MMEF% predicted** | | |
| --- | --- | --- | --- | --- | --- | --- | --- |
|  | **Estimate** | **P value** | **95% CI** |  | **Estimate** | **P value** | **95% CI** |
| **Intercept** | **10.592** | **<.001** | **7.684, 13.500** |  | **-24.311** | **<.001** | **-27.220, -21.403** |
| **No. of bronchiectatic lobes** | **0.675** | **.011** | **0.157, 1.194** |  | **-7.645** | **<.001** | **-8.163, -7.126** |
| **HRCT total score*** | -0.172 | .343 | -0.527, 0.184 |  | **5.918** | **<.001** | **5.563, 6.274** |
| **Age** | **0.078** | **<.001** | **0.054, 0.102** |  | **-0.260** | **<.001** | **-0.283, -0.236** |
| **FEV_1_ predicted%** | **-0.039** | **.001** | **-0.062, -0.016** |  | **1.397** | **<.001** | **1.374, 1.420** |
| **Sex** | - | - | - |  | - | - | - |
| Males | Reference | Reference | Reference |  | Reference | Reference | Reference |
| Females | **1.044** | **.006** | **0.303, 1.785** |  | **-13.443** | **<.001** | **-14.184, -12.702** |
| **Sputum bacteriology** | - | - | - |  | - | - | - |
| *P. aeruginosa* colonized | Reference | Reference | Reference |  | Reference | Reference | Reference |
| No *P. aeruginosa* colonized | **1.220** | **.010** | **0.290, 2.149** |  | **-15.944** | **<.001** | **-16.873, -15.014** |
| **Cystic bronchiectasis** | - | - | - |  | - | - | - |
| Yes | Reference | Reference | Reference |  | Reference | Reference | Reference |
| No | -0.280 | .476 | -1.051, 0.491 |  | **1.572** | **<.001** | **0.801, 2.344** |
| **Heterogeneity** | - | - | - |  | - | - | - |
| Yes | Reference | Reference | Reference |  | Reference | Reference | Reference |
| No | **-1.371** | **<.001** | **-2.082, -0.660** |  | **1.654** | **<.001** | **0.943, 2.365** |
| **Bilateral bronchiectasis** | - | - | - |  | - | - | - |
| Yes | Reference | Reference | Reference |  | Reference | Reference | Reference |
| No | -0.407 | .295 | -1.168, 0.355 |  | **3.737** | **<.001** | **2.976, 4.498** |

95%CI: 95% confidence interval, LCI: lung clearance index; MMEF: maximal mid-expiratory flow

Data in bold indicated the statistical analyses with significance.

*Modified Reiff score

**Table S15 Fixed-effect estimates in multivariate linear mixed model of the clinical variable attributes’ impacts on lung clearance index and maximal mid-expiratory flow in patients with HRCT score equal to or greater than 7 and lower than 13**

|  | **LCI** | | |  | **MMEF% predicted** | | |
| --- | --- | --- | --- | --- | --- | --- | --- |
|  | **Estimate** | **P value** | **95% CI** |  | **Estimate** | **P value** | **95% CI** |
| **Intercept** | **18.553** | **<.001** | **14.862, 22.204** |  | **-19.509** | **<.001** | **-23.180, 15.838** |
| **No. of bronchiectatic lobes** | -0.033 | .868 | -0.423, 0.357 |  | **1.388** | **<.001** | **0.998, 1.778** |
| **HRCT total score*** | **0.356** | **.037** | **0.021, 0.690** |  | **-1.852** | **<.001** | **-2.186, -1.517** |
| **Age** | 0.017 | .171 | -0.007, 0.041 |  | **0.334** | **<.001** | **0.310, 0.359** |
| **FEV_1_ predicted%** | **-0.122** | **<.001** | **-0.140, -0.103** |  | **0.914** | **<.001** | **0.896, 0.932** |
| **Sex** | - | - | - |  | - | - | - |
| Males | Reference | Reference | Reference |  | Reference | Reference | Reference |
| Females | 0.614 | .089 | -0.093, 1.321 |  | **4.905** | **<.001** | **4.198, 5.612** |
| **Sputum bacteriology** | - | - | - |  | - | - | - |
| *P. aeruginosa* colonized | Reference | Reference | Reference |  | Reference | Reference | Reference |
| No *P. aeruginosa* colonized | -0.484 | .190 | -1.208, 0.239 |  | **-1.216** | **.001** | **-1.940, -0.493** |
| **Cystic bronchiectasis** | - | - | - |  | - | - | - |
| Yes | Reference | Reference | Reference |  | Reference | Reference | Reference |
| No | **1.780** | **<.001** | **0.928, 2.631** |  | **-7.208** | **<.001** | **-8.060, -6.357** |
| **Heterogeneity** | - | - | - |  | - | - | - |
| Yes | Reference | Reference | Reference |  | Reference | Reference | Reference |
| No | 0.317 | .424 | -0.460, 1.094 |  | **-3.729** | **<.001** | **-4.506, -2.953** |
| **Bilateral bronchiectasis** | - | - | - |  | - | - | - |
| Yes | Reference | Reference | Reference |  | Reference | Reference | Reference |
| No | -0.310 | .787 | -2.568, 1.947 |  | **-17.709** | **<.001** | **-19.966, -15.452** |

95%CI: 95% confidence interval, LCI: lung clearance index; MMEF: maximal mid-expiratory flow

Data in bold indicated the statistical analyses with significance.

*Modified Reiff score

**Table S16** Fixed-effect estimates in multivariate linear mixed model of the clinical variable attributes’ impacts on lung clearance index and maximal mid-expiratory flow in patients with HRCT total score ≥13

|  | **LCI** | | |  | **MMEF% predicted** | | |
| --- | --- | --- | --- | --- | --- | --- | --- |
|  | **Estimate** | **P value** | **95% CI** |  | **Estimate** | **P value** | **95% CI** |
| **Intercept** | **35.226** | **<.001** | **24.800, 45.652** |  | **-52.359** | **<.001** | **-62.785, -41.933** |
| **No. of bronchiectatic lobes** | NA | NA | NA |  | -0.247 | .749 | -1.761, 1.268 |
| **HRCT total score*** | -0.192 | .307 | -0.560, 0.176 |  | **2.804** | **<.001** | **2.436, 3.172** |
| **Age** | **0.172** | **<.001** | **0.130, 0.213** |  | **-0.091** | **<.001** | **-0.132, -0.049** |
| **FEV_1_ predicted%** | **-0.060** | **<.001** | **-0.089, -0.031** |  | **0.889** | **<.001** | **0.860, 0.918** |
| **Sex** | - | - | - |  | - | - | - |
| Males | Reference | Reference | Reference |  | Reference | Reference | Reference |
| Females | **1.345** | **.014** | **0.269, 2.420** |  | **5.529** | **<.001** | **4.454, 6.605** |
| **Sputum bacteriology** | - | - | - |  | - | - | - |
| *P. aeruginosa* colonized | Reference | Reference | Reference |  | Reference | Reference | Reference |
| No *P. aeruginosa* colonized | **-2.925** | **<.001** | **-3.936, -1.914** |  | 0.283 | .583 | -0.728, 1.294 |
| **Cystic bronchiectasis** | - | - | - |  | - | - | - |
| Yes | Reference | Reference | Reference |  | Reference | Reference | Reference |
| No | **-4.994** | **<.001** | **-7.378, -2.609** |  | **-5.487** | **<.001** | **-7.871, -3.102** |
| **Heterogeneity** | - | - | - |  | - | - | - |
| Yes | Reference | Reference | Reference |  | Reference | Reference | Reference |
| No | NA | NA | NA |  | NA | NA | NA |
| **Bilateral bronchiectasis** | - | - | - |  | - | - | - |
| Yes | Reference | Reference | Reference |  | Reference | Reference | Reference |
| No | NA | NA | NA |  | NA | NA | NA |

95%CI: 95% confidence interval, LCI: lung clearance index; MMEF: maximal mid-expiratory flow

Data in bold indicated the statistical analyses with significance.

*Modified Reiff score

NA: not applicable

**Figure legend**

**
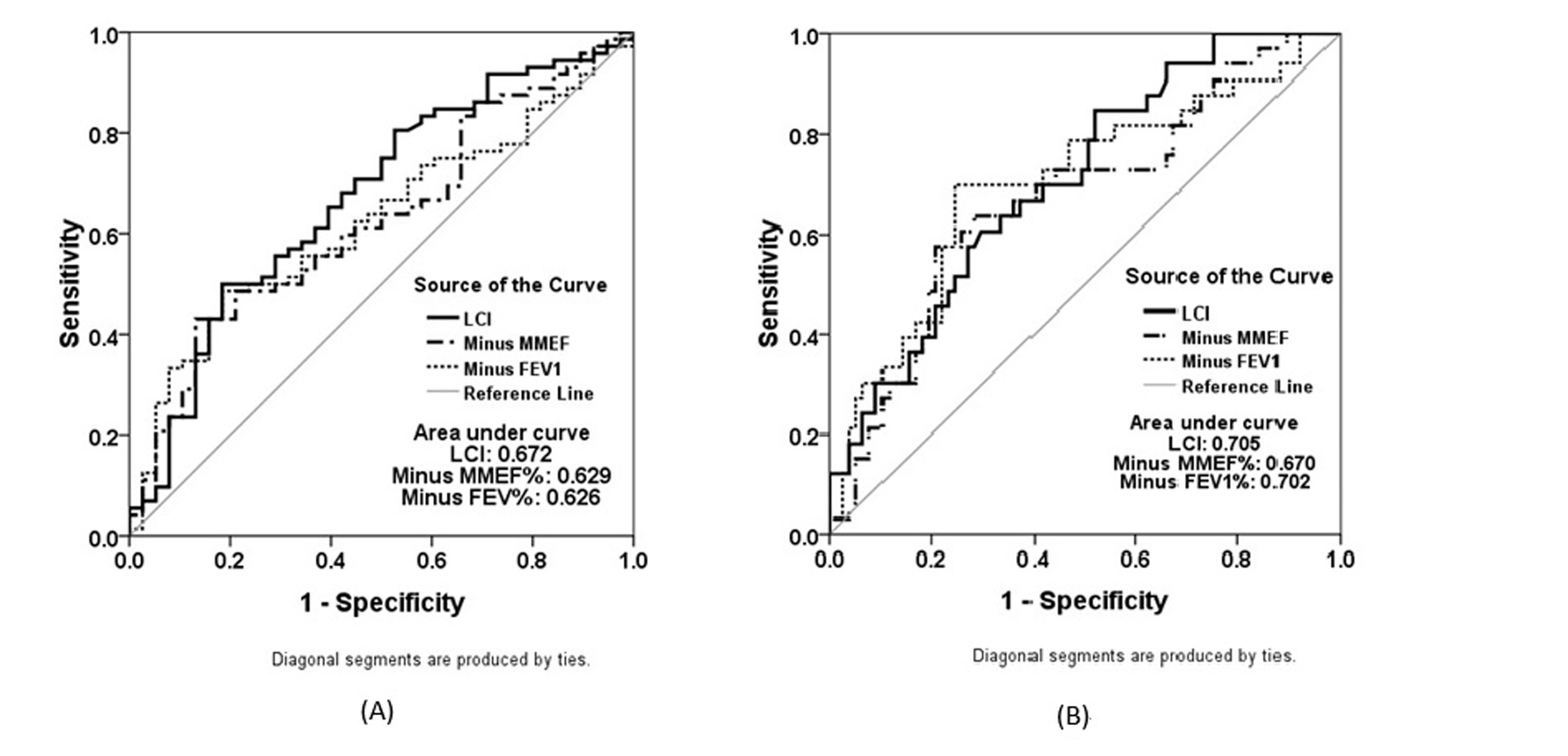
**

**Figure E1. The diagnostic performance of LCI, MMEF% predicted and FEV_1_% predicted in patients with bronchiectasis**

Figure E1-A. The diagnostic performance of LCI, MMEF% predicted and FEV_1_% predicted for discriminating patients with moderate-to-severe bronchiectasis from those with mild bronchiectasis;

Figure E1-B. The diagnostic performance of LCI, MMEF% predicted and FEV_1_% predicted for discriminating patients with severe bronchiectasis from those with mild-to-moderate bronchiectasis.
